# Improving the impact of HIV pre-exposure prophylaxis implementation in small urban centers among men who have sex with men: An agent-based modelling study

**DOI:** 10.1371/journal.pone.0199915

**Published:** 2018-07-09

**Authors:** Jason R. Gantenberg, Maximilian King, Madeline C. Montgomery, Omar Galárraga, Mattia Prosperi, Philip A. Chan, Brandon D. L. Marshall

**Affiliations:** 1 Department of Epidemiology, Brown University School of Public Health, Providence, RI, United States of America; 2 Department of Medicine, Brown University, Providence, RI, United States of America; 3 Department of Health Services, Policy, and Practice, Brown University School of Public Health, Providence, RI, United States of America; 4 Department of Epidemiology, College of Public Health and Health Professions & College of Medicine, University of Florida, Gainesville, FL, United States of America; Emory University School of Public Health, UNITED STATES

## Abstract

**Objectives:**

Identifying prescribing strategies that improve the efficiency of PrEP should increase its impact at the population level. This study identifies PrEP allocation criteria that most effectively reduce 10-year HIV incidence by 25%, in accordance with the US National HIV/AIDS Strategy’s goal for the proportionate reduction in new diagnoses.

**Methods:**

We used a discrete-time stochastic agent-based model to simulate several PrEP engagement strategies. The model represented MSM aged 15–74 in Rhode Island and was calibrated to statewide prevalence from 2009–2014. We simulated HIV transmission in the absence of PrEP and compared the following PrEP engagement scenarios: 1) allocation to the current patient population; 2) random allocation; 3) allocation to MSM with greater than 5 sexual partners in one year; 4) allocation to MSM with greater than 10 sexual partners in one year.

For each scenario and coverage level we estimated the number and proportion of infections averted and the person-years on PrEP per averted infection.

**Results:**

In 2014, HIV prevalence before PrEP implementation was between 4% and 5%. In the *No PrEP* scenario 826 new infections (95% simulation limits [SL]: 711, 955) occurred over 10 years, with an incidence rate of 3.51 per 1000 person-years (95% SL: 3.00, 4.08). Prevalence rose to 7.4% (95% SL: 6.7, 8.1). None of the PrEP scenarios reduced new HIV infections by 25% while covering less than 15% of the HIV-uninfected population. At 15% coverage, allocating PrEP to the current patient population, MSM with greater than 5 sexual partners in a year, and MSM with greater than 10 partners reduced new infections by at least 25%, requiring 161 (95% SL: 115, 289), 150 (95% SL: 107, 252), and 128 (95% SL: 100, 184) person-years on PrEP per averted infection, respectively.

**Conclusions:**

Engaging MSM with high numbers of sexual partners would improve the population-level impact and efficiency of PrEP in settings where PrEP coverage remains low. However, the sustained population-level PrEP coverage needed to reduce new infections by 25% is substantially higher than current levels of PrEP uptake.

## Introduction

While HIV incidence among men who have sex with men (MSM) in the United States has stabilized over the past several years [1], MSM account for approximately 70% of new infections [2]. Moreover, in 2010, the National HIV/AIDS Strategy (NHAS) set a 10-year goal of reducing the number of new HIV diagnoses in the US by 25% [3]. Given current trends, at the start of 2020, these goals will be unlikely to have been met among MSM populations, but the Strategy identifies expanded pre-exposure prophylaxis (PrEP) use as one of the primary biomedical interventions among those recommended for mitigating the epidemic in the United States.

Randomized controlled trials and demonstration projects have established the efficacy and effectiveness of pre-exposure prophylaxis (PrEP) for the prevention of HIV in MSM [4–8], yet PrEP uptake overall appears to have been slow to date in most areas of the US, despite widespread eligibility [9]. Modelling studies have suggested that a more robust scale-up will be required to realize significant reductions in incidence at the population level, even in areas with high background HIV incidence among MSM [10–12]. To our knowledge, however, no simulation studies have evaluated statewide PrEP implementation, and while other authors have used data from PrEP demonstration projects and trials to inform relevant parameters, we are unaware of any studies that have used primary clinic data endogenous to the target population to inform adherence and sexual behavior parameters among MSM on PrEP. Furthermore, as most of the aforementioned analyses have focused on HIV epidemics among MSM in large urban centers [10,11], we believe that modelling smaller urban settings like Rhode Island, where the epidemic is typified by generally lower prevalence, may be useful for informing PrEP implementation in these areas [13,14].

Given the continued uncertainty surrounding the feasibility and ultimate reach of PrEP scale-up, determining allocation strategies that maximize both the population-level impact and efficiency of PrEP will be important to informing ongoing and future implementation efforts in a range of settings. These same modelling studies have also suggested differential efficiency of PrEP by risk group [10–12]. Jenness et al, for instance, simulated the effect of the Center for Disease Control and Prevention’s recommended PrEP indications in MSM [15] and found that allocation to individuals within serodiscordant partnerships averted a large number of infections while minimizing the number needed to treat [11]. The authors also found that allocation to those engaging in condomless anal sex within HIV-status-unknown partnerships achieved a similar population-level reduction in new HIV cases but did so less efficiently [11].

To inform statewide PrEP implementation policies, we sought to compare hypothetical PrEP allocation scenarios to determine which of these maximized PrEP impact (i.e., reductions in population-level HIV incidence) and efficiency over 10 years. In particular, we sought to identify scenarios that would most efficiently achieve the 2020 NHAS goals of producing a 25% reduction in incidence [3]. To achieve these objectives, we used patient data from a statewide PrEP clinic to parameterize an agent-based model (ABM) that simulates HIV transmission in a dynamic sexual network of MSM.

## Methods

### Model overview

We constructed a discrete-time stochastic ABM to simulate HIV transmission within a virtual population of agents (*N* = 25,000) parameterized to represent all MSM aged 15–74 in Rhode Island. Agents in the model were assigned both fixed and variable characteristics related to demographic attributes, sexual behavior, HIV status, antiretroviral treatment (ART) resulting in viral suppression, and PrEP. Fixed characteristics included sexual role preference (insertive-only, receptive-only, versatile), mean number of annual sex partners (actual partner number varied annually), and mean annual sex frequency per partner. Time-updated attributes included age, HIV status, PrEP status, and viral suppression (yes/no). To keep the population size static, agents who died were replaced by new HIV-negative agents from the same age group. For all other characteristics, agents “born” into the model were assigned characteristics stochastically according to the seed distributions. More information regarding model processes and parameterization is available in Table 1 and S1 Appendix.

**Table 1:** Overview of model parameters and processes.

Processes	Provenance	Sources
***Demography***		
Population size	Rhode Island	[16,17]
Age (15–74)	Rhode Island	[18]
Background mortality	Rhode Island	[19]
***Sexual Behavior***		
Condom use	External	[20]
Sexual role	External	[21]
***Sexual Networks***		
Annual partner number	Rhode Island Selection into clinic (Rhode Island, primary data)	[22]
Sex frequency	External	[23]
Relationship duration	External	[23]
Assortative mixing	External, Assumed	Based on [10,24,25]
***HIV/AIDS***		
Testing probability	Rhode Island, calibrated	Starting probability from [22]
Proportion of PLWH diagnosed	Rhode Island	[14]
Proportion of PLWH on ART	Rhode Island	Calculated, [26]
Proportion of PLWH virally suppressed	Rhode Island	[13,27]
Proportion of PLWH currently with AIDS	Rhode Island	Inferred, based on [28]
Transmission probability by sexual position	External	[29]
Transmission risk reduction due to HIV diagnosed status	Assumed	Assumed
Transmission risk reduction due to viral suppression	External	[29]
HIV prevalence (age-specific)	Rhode Island	[30]
HIV/AIDS-related mortality rate ratio	External, Rhode Island	[13,14,27,28,31–33]
***Pre-exposure Prophylaxis***		
Dropout rate	Rhode Island	[34]
Adherence probability (full vs. partial)	Rhode Island	[34]
HIV transmission risk reduction conferred by full and partial adherence	External	[5]

PLWH, people living with HIV; PrEP, pre-exposure prophylaxis; ART, antiretroviral therapy. Parameters, their values, and their sources are discussed in more detail in S1 Appendix.

### Sexual partnerships

Agents were assigned a static mean annual partner number which determined the expectation value for the mean number of sexual partners for a given year. Each year, the agent drew an annual target partner number from their static distribution and achieved this target probabilistically. In this way, agents can be considered to have had sexual tendencies with regard to partner acquisition while exhibiting behavioral variation from year to year.

During each time step, agents searched for and acquired partners stochastically. New partnerships were assigned a duration based on distributions described in a study by Wall et al [23]. The model prohibited certain sexual partnerships based on role preference—for instance, two exclusively insertive agents could not pair. To account for assortative mixing by age, agents had a 50% probability of pairing within their own age group; the probability of pairing with agents outside an index agent’s age group decreased as a function of age discordance (see S1 Appendix). At the dyadic level, the number of sex acts was assigned as an average between each individual agent’s desired annual per-partnership sexual frequency [23], a fixed attribute assigned to each agent at initialization.

Each month, agents engaged in a given number of sex acts with their partners, probabilistically transmitted HIV within serodiscordant partnerships, and initiated or discontinued PrEP or HIV treatment. The model simulated at-risk sexual contacts, defined as condomless anal sex acts within serodiscordant partnerships. The probability of condom use during a given sexual episode was based on the number of prior contacts between two paired agents, derived from data regarding the most recent sexual episode among a sample of MSM [20]. Per-act HIV transmission risk within HIV serodiscordant couples was modified directly by several factors: 1) the PrEP status of the uninfected partner, 2) the respective sexual roles of the infected and uninfected agents, 3) the viral suppression status of the infected agent, and 4) the HIV-positive partner’s diagnosis status.

### HIV testing and treatment

HIV-uninfected agents tested for HIV with a starting frequency based on Rhode Island data [22], and this value was tuned such that approximately 82% were diagnosed [14]. Diagnosed agents initiated ART stochastically, and a subset of these become virally suppressed, with a target rate of viral suppression among HIV-infected agents of 45% [27] (see S1 Appendix). Independent of treatment status, sexual acts involving HIV-infected agents aware of their infection were subject to a decreased risk of transmission based on the general observation that HIV-infected MSM reduce certain risk behaviors post-diagnosis [35–38]. For serodiscordant partnerships in which the HIV-infected agent was diagnosed, the overall transmission probability to the HIV-uninfected partner was scaled by a fixed value of 0.5.

### Model calibration

The model was calibrated to reproduce the inferred HIV prevalence in Rhode Island between 2008 and 2014 along with new diagnoses, based on surveillance data from the National Center for HIV/AIDS, Viral Hepatitis, STD, and TB Prevention Atlas [30]. We inferred the true HIV prevalence, assuming that in each year, prevalent diagnosis in the NCHHSTP Atlas represented 82% of the true number of cases [14]. See S1 Fig for a summary of relevant calibration targets. Additional targets included the proportion of HIV-infected agents virally suppressed, the proportion diagnosed, and age-specific HIV incidence/diagnoses.

Each run progressed from a stochastically generated base population, the probability of prevalent HIV infection at model initialization for a given agent being conditional upon their age (see S1 Appendix). After calibration, each model run incorporated a burn-in period of 6 years to recreate the HIV prevalence trend in Rhode Island, after which PrEP was introduced and its effects on HIV transmission observed from 2015 through 2024.

### PrEP scenarios

The following PrEP scenarios were modelled, in which PrEP was preferentially allocated to MSM based on particular characteristics deemed to be of interest:

1. *No PrEP*—PrEP was never implemented
2. *Current Patient Population*—Agents who initiated PrEP did so based on the partner number and age distributions of the Miriam Hospital PrEP Clinic
3. *Random*—PrEP agents were selected at random from the HIV-uninfected population
4. *Annual Partner Number*
  - Agents were selected for PrEP if they had a target annual partner number of greater than 5 (*PN* > 5) in the current 12-month interval
  - Agents were selected for PrEP if they had a target annual partner number of greater than 10 (*PN* > 10) in the current 12-month interval

In all cases, PrEP was implemented across a range of population coverage rates between 0 and 30%, defined as the proportion of all HIV-uninfected MSM on PrEP. In each PrEP allocation scenario, the predetermined population-level PrEP coverage was achieved by administering prescriptions only to those agents who met the scenario’s criteria. For example, covering 10% of the entire HIV-uninfected MSM population was achieved either by allocating all available prescriptions each month to agents meeting the CPP criteria or, alternatively, to those who had drawn a target of at least 5 or 10 partners for a given year during simulation, depending on the scenario in play. The maximum population-level coverage in PrEP-targeted scenarios was therefore limited in some cases by the size of the indicated subpopulation. Random allocation to all negative MSM was included as a referent (i.e., “control”) scenario to assess whether expanding PrEP to patients based on the current patient population might perform better than randomly in this context.

We elected to impose the PrEP coverage level immediately upon implementation in the model and maintain that proportion over the course of the simulation. During each timestep, a predetermined number of prescriptions were made available. Agents who dropped out of PrEP were replaced by new agents whose probability of selection varied based on the particular engagement scenario.

### The Miriam Hospital PrEP clinic

The Miriam Hospital (TMH) is the state’s primary provider of PrEP and other HIV-related clinical services. Information regarding the early implementation of the PrEP clinic at TMH has been published previously [39,40]. In 2013, the clinic began prescribing PrEP to patients who meet criteria set forth in national CDC guidelines [15]. The PrEP *Current Patient Population* allocation scenario used information from a row-level dataset describing the first 241 MSM to initiate PrEP at this clinic. In this scenario, agents initiated PrEP based on quintiles of the observed annual sexual partner number distribution and an age distribution grouped as follows: 15–24, 25–34, 35–44, 45–54, 55+. These distributions were treated as independent during selection for PrEP in the simulations. In all scenarios, rates of agent adherence were based on early data from this cohort of TMH PrEP clinic patients [34]; based on this data, 82% of PrEP agents were classified as “fully adherent, 4+ doses/week”, corresponding to a 96% reduction in HIV transmission risk, and the remainder as “partially adherent”, corresponding to a 76% reduction, in accordance with data on the association between drug concentrations and protection against HIV acquisition [5]. Retention was translated into a monthly probability of a PrEP agent’s discontinuing their prescription, based on clinical data from The Miriam Hospital PrEP Clinic patients (described in more detail in S1 Appendix). We applied these adherence and dropout rates to all PrEP allocation scenarios.

### Analysis

Each scenario was simulated 1000 times. To characterize the distribution of model outputs, we present medians and 95% simulation limits.

We calculated 10-year incidence rates and cumulative incidence, the number/proportion of infections averted (NIA/PIA, respectively), and the person-years on PrEP per averted infection (PYPAI). NIAs were calculated by subtracting the 10-year cumulative incidence for each of the 1000 runs within a given PrEP allocation scenario from the mean cumulative incidence in the No PrEP scenario, following a method based on that of Jenness et al [11]. PYPAI was calculated by summing the total person-years on PrEP within the allocation scenario and dividing by the NIA. We penalized the PYPAI distribution to account for instances in which transmission within a PrEP allocation scenario run exceeded mean incidence in the No PrEP scenario and thus led to a negative or null NIA: for any scenario in which the NIA was ≤ 0, we set the denominator to 1 when calculating the PYPAI. Doing so avoided including observations in a way that would have artificially improved the efficiency measure.

### Sensitivity analysis

We performed a number of sensitivity analyses to examine the effects of perturbing parameters one at a time. The following alternative scenarios were simulated:

1. Population sexual frequency (+ 50% / - 50%)
2. Target annual partner number (+ 50% / - 50%)
3. PrEP adherence among agents in care (full vs. partial)
  - 0% vs. 100%
  - 50% vs. 50%
  - 100% vs. 0%
4. Assortative mixing proportions
  - Random mixing by age
  - Use of an age mixing matrix specifying a 50% probability of acquiring a within-group partner for 0–75% of partner selections
  - Use of an age mixing matrix specifying a 50% probability of acquiring a within-group partner for 100% of partner selections (used in main analysis)

Because scenarios 1, 2, and 4 would have changed underlying transmission dynamics in the absence of PrEP by altering the rate of at-risk contacts and/or sexual network density, we ran separate base-case models at 0% PrEP coverage for each of these sensitivity analyses. Subsequently, we simulated the *Current Patient Population* allocation scheme at 15% PrEP coverage using the corresponding base-case as a reference for calculating NIA, PIA, and PYPAI. For scenario 3, we modelled different PrEP adherence rates at 15% coverage and compared outcomes against the same 0% PrEP coverage simulations used in the main analysis.

### Ethics statement

The institutional review boards at The Miriam Hospital and Brown University approved this study (#1603001437). Patients provided written informed consent to take part in an observational cohort from which the clinic data for this study was derived.

## Results

In the absence of PrEP implementation, the model predicted a median of 826 infections over 10 years (95% SI: 711–955) and an aggregate incidence rate of 3.51 per 1000 person-years at risk (95% SI: 3.00–4.08). At the beginning of the simulation (2008), HIV prevalence was approximately 3.5% and rose to 4.7% (95% SI: 4.3–5.1) by the beginning of the analytic window (2015). HIV prevalence at the end of 2024 in the base case was 7.4% (95% SI: 6.7–8.1).

At 15% PrEP coverage, most scenarios reduced HIV incidence by at least 25%, with the exception of the *Random* scenario. At 20% coverage of the HIV-uninfected population, all scenarios achieved a 25% reduction in incidence. No scenario averted 25% of infections at lower PrEP coverage, though allocation to MSM with greater than 10 partners reduced infections by a median of 23.5% (95% SI: 10.9–34.3) at 10% coverage over 10 years. (See Table 2 and S2 Fig).

**Table 2:** Ten-year summary statistics across PrEP allocation scenarios (15% coverage) vs. base case (0% PrEP coverage).

Scenario	HIV Prevalence (%)	New Infections	Incidence Rate*	Infections Averted (#)	Infections Averted (%)	PYPAI
No PrEP	7.4 (6.7, 8.1)	826 (711, 955)	3.51 (3.00, 4.08)	-	-	-
Current patients	6.5 (5.9, 7.2)	612 (523, 709)	2.59 (2.21, 3.02)	218 (121, 307)	26.2 (14.5, 37.0)	161 (115, 289)
Random	6.7 (6.0, 7.4)	654 (546, 756)	2.77 (2.31, 3.22)	176 (74, 284)	21.2 (8.9, 34.2)	199 (124, 474)
PN > 5	6.5 (5.8, 7.1)	595 (499, 691)	2.52 (2.11, 2.94)	235 (139, 331)	28.3 (16.7, 39.9)	150 (107, 252)
PN > 10	6.3 (5.7, 6.9)	555 (478, 639)	2.35 (2.01, 2.71)	275 (191, 352)	33.1 (23.0, 42.4)	128 (100, 184)

**Notes:** *HIV Prevalence*, ending HIV prevalence; *PYPAI*, person-years on PrEP per averted infection; *Current patients*, Current Patient Population scenario; *PN > 5*, expected annual partner number greater than 5; *PN > 10*, expected annual partner number greater than 10. Medians and 95% simulation limits presented.

* Incidence rate per 1000 person-years at risk.

**Fig 1:**
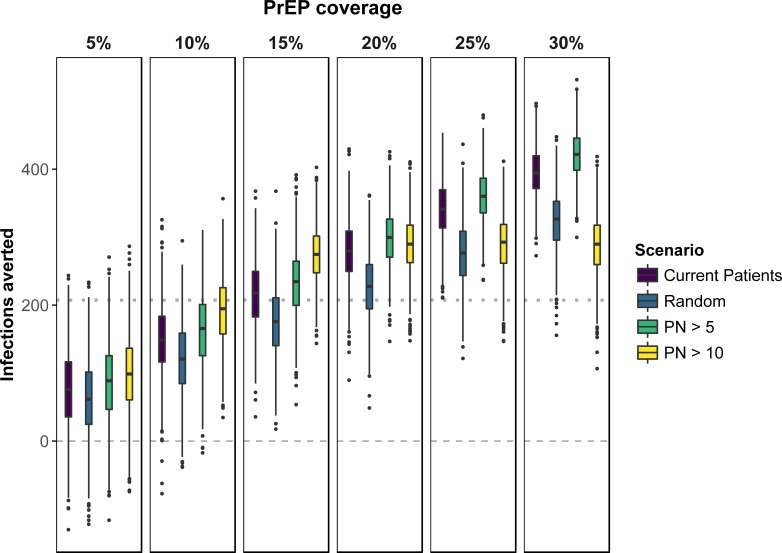
Distribution of NIA (number of infections averted) estimates produced by 1000 independent simulations for each PrEP allocation scenario. Mean cumulative incidence in base case scenario used as reference to calculate NIA. *Dashed line*, null effect of PrEP; *Dotted line*, 25% reduction in cumulative incidence. Negative values indicate individual PrEP scenario runs in which cumulative incidence exceeded the mean cumulative incidence in the No PrEP scenario. (Note: The PN > 10 allocation scenario covered a maximum of approximately 15–17% of the HIV-uninfected population. PrEP coverage scenarios between 20% and 30% should be interpreted with this ceiling coverage in mind when comparing allocation criteria against one another).

**Fig 2:**
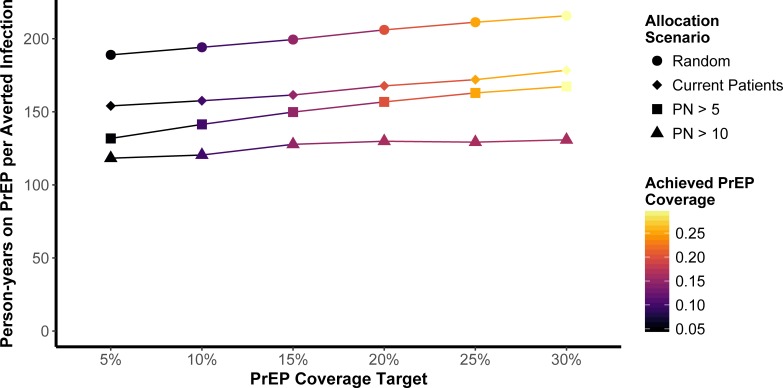
Median person-years on PrEP per averted infection (PYPAI) by PrEP coverage target and actual achieved PrEP coverage across each of the allocation scenarios. Achieved PrEP coverage refers to the actual simulated proportion of all HIV-uninfected MSM on PrEP for each coverage and allocation scenario. Because PYPAI outcome distributions were heavily right-skewed at lower PrEP coverages—due in part to how the measure was penalized to account for runs in which PrEP scenarios featured more infections than the mean base case—simulation intervals are omitted to avoid overplotting.

The *Current Patient Population* scenario appeared to increase the impact of PrEP over that of random allocation, as measured by the NIA. Provision of PrEP to agents with high mean annual partner numbers (greater than 5 or greater than 10) improved both PrEP effectiveness and reduced the number of person-years on PrEP per averted infection (Figs 1 and 2). The impact of allocation to MSM with annual partner numbers greater than 10 is limited at higher PrEP coverage due to a smaller number of MSM with this characteristic.

Results pertaining to age-specific outcomes and PrEP allocation are presented in brief within S1 Appendix.

### Sensitivity analyses

**Fig 3:**
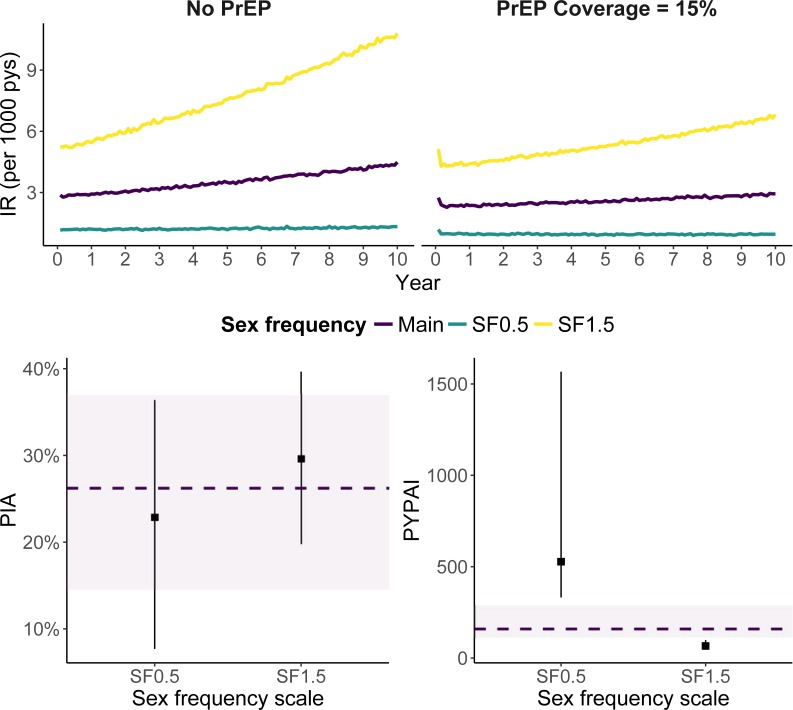
HIV incidence rates, PIA, and PYPAI by population sex frequency scenario. Each scenario scaled sex frequency by the factors depicted in the images. In the top row, lines encode means at each year. In the bottom row, the dashed line and purple band indicate the median and 95% simulation interval in the *Current Patients* PrEP allocation scenario at 15% coverage. Point ranges depict these statistics for the alternative sex frequency scenarios, also at 15% PrEP coverage. *Abbreviations*: IR, incidence rate per 1000 person-years at risk; PIA, percentage of infections averted; PYPAI, person-years per averted infected.

Scaling the annual anal sex frequency distribution to investigate PrEP measures in settings with differing incidence indicated similar proportional reductions in HIV incidence due to PrEP compared to the main analysis (Table 3, Fig 3). PrEP efficiency, however, improved by 59% relative to the base case when sex frequency was scaled upward by 50%. This reflects a drop in the median PYPAI from 161 to 66 (Table 4). Conversely, halving sex frequency resulted in a rise of the PYPAI to 527, a 227% increase. Changes to the annual partner number distribution using the same proportional scales resulted in significantly more pronounced changes to epidemiologic outputs and measures of PrEP impact and efficiency when scaled upward (Tables 3 and 4).

**Table 3:** Sex frequency and partner number sensitivity analyses. HIV prevalence and incidence at 0% PrEP coverage.

Scenario	HIV Prevalence	Cumulative Incidence	Incidence Rate	Incidence Change (%)
Main	7.4 (6.7, 8.1)	826 (711, 955)	3.51 (3.00, 4.08)	-
*Sex Frequency*				
SF0.5	4.5 (4.1, 4.9)	294 (246, 348)	1.23 (1.03, 1.46)	-64.3
SF1.5	11.9 (10.7, 13.3)	1756 (1516, 2003)	7.65 (6.56, 8.81)	112.6
*Partner Number*				
PN0.5	5.2 (4.7, 5.7)	389 (332, 459)	1.63 (1.39, 1.93)	-52.9
PN1.5*	24.1 (21.8, 26.4)	4487 (4011, 4930)	20.88 (18.39, 23.30)	443.2

**Notes:**

*Main*, base case from main analysis

*SF[X]*, sex frequency scale

*PN[X]*, partner number scale

*HIV Prevalence*, ending HIV prevalence; *CumInc*, new infections over 10 years; *IR*, incidence rate per 1000 person-years at risk

Incidence change = percent change in 10-year median cumulative incidence relative to Main scenario

Medians and 95% simulation limits reported

* Omits 62 runs for which submitted jobs timed out (*N* = 938 independent simulations). All other results based on 1000 independent simulations for each scenario.

**Table 4:** Sex frequency, partner number, and PrEP adherence sensitivity analyses. PrEP impact and efficiency at 15% coverage of HIV-negative MSM.

Scenario	NIA	PIA	PYPAI
Main	218 (121, 307)	26.2 (14.5, 37.0)	161 (115, 289)
*Sex Frequency*			
SF0.5	68 (23, 108)	22.9 (7.7, 36.4)	527 (332, 1567)
SF1.54	520 (348, 696)	29.6 (19.8, 39.7)	66 (50, 98)
*Partner Number*			
PN0.5	89 (34, 142)	22.7 (8.6, 36.2)	401 (251, 1059)
PN1.5*	1471 (1134, 1809)	32.8 (25.3, 40.4)	23 (19, 29)
*PrEP Adherence*			
Adh0.00	190 (79, 285)	22.9 (9.5, 34.3)	185 (124, 444)
Adh0.50	202 (100, 295)	24.4 (12.0, 35.5)	174 (120, 350)
Adh1.00	221 (125, 316)	26.6 (15.0, 38.0)	159 (112, 281)

**Notes:**

*Main*, base case from main analysis

*SF[X]*, sex frequency scale

*Adh[X]*, proportion of PrEP agents fully adherent (remainder are partially adherent in all cases)

*PrEP*, pre-exposure prophylaxis; *MSM*, men who have sex with men; *NIA*, number of infections averted, *PIA*, percentage of infections averted; *PYPAI*, person-years per averted infection

Medians and 95% simulation limits reported

* Omits 40 runs for which submitted jobs timed out (*N* = 960 independent simulations). All other results based on 1000 independent simulations for each scenario.

**Fig 4:**
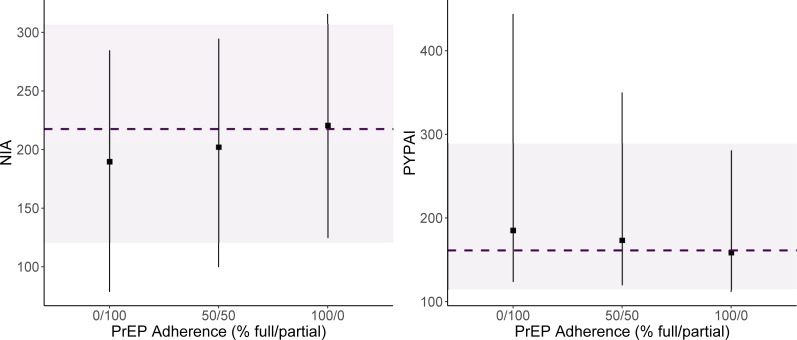
NIA and PYPAI measures by PrEP adherence scenario. Each scenario altered the proportions of individuals on PrEP who were fully vs. partially adherent (0/100, 50/50, 100/0, respectively). In the main analysis, 82% of current PrEP patients were considered to be fully adherent. NIA, number of infections averted; PYPAI, person-years per averted infection. The dashed line and purple band indicate the median and 95% simulation interval in the *Current Patients* PrEP allocation scenario at 15% coverage. Point ranges indicate these same statistics for the alternative PrEP adherence scenarios, also at 15% coverage.

Altering the proportion of active PrEP agents who are fully adherent did not result in changes in PrEP-related measures, with minimal changes to median NIA and PYPAI (Table 4, Fig 4). Additional comments on this sensitivity analysis appear in the S1 Appendix.

Use of the age mixing matrix for 100% of partnership acquisitions resulted in a pattern of incidence across age groups that most closely approached the observed targets. The effects of partial use of the matrix (0%–75% of the time) are displayed in the S1 Appendix (S3 Fig).

## Discussion

In this study, we estimated the population impact of varying PrEP allocation strategies on HIV prevalence and incidence among MSM at a state level. We found that a significant increase in PrEP coverage, in addition to the targeted engagement of HIV-uninfected MSM with higher numbers of partners in a PrEP program, could result in 10-year decreases in HIV incidence that would meet the National HIV/AIDS Strategy goals, though not by 2020.

We found that a scenario in which PrEP continues to be provided to individuals who mirror the current PrEP clinic population is more efficient and effective than random PrEP allocation. This finding is consistent with a modelling study demonstrating that the Centers for Disease Control and Prevention (CDC) guidelines strike a reasonable balance between epidemiological impact and program efficiency [11]. In our study, we did not model these guidelines explicitly, though selecting agents for PrEP based on the empirical partner number distribution from a real-world clinic accounts for likely the most important risk factor for HIV acquisition. Hypothetical scenarios engaging agents with higher numbers of partners tended to improve both PrEP impact and efficiency. Notably, PrEP allocation to agents with greater than 10 partners in a year averted 33% of HIV infections over 10 years at 15% coverage of the HIV-negative population. Kasaie et al found an approximately 20% decrease in HIV incidence over 5 years when allocating PrEP to MSM with greater than 5 partners annually, which achieved approximately 13% coverage of HIV-negative population [10]. Both Jenness et al and Kasaie et al modelled PrEP within higher incidence settings (Atlanta and Baltimore, respectively), whereas our study extends the prior PrEP modelling literature by simulating statewide program implementation in a target population with lower HIV prevalence and incidence. Because implications for efficient PrEP implementation may vary by setting [41], we believe comparing results from modelling studies using different parameters and assumptions can be informative for policy and public health practice.

Care should be taken in interpreting reductions in HIV incidence directly, as we implemented PrEP coverage at the specified level immediately, without imposing a scale-up period; therefore, these figures cannot be considered true forecasts. Nevertheless, our results provide some guidance in early-stage PrEP implementation, either at the state or local level. Engagement and increased uptake of PrEP among MSM with high numbers of partners could result in earlier population-level impact and better efficiency. However, priorities and strategies for implementation may to be setting-specific if underlying epidemiology and sexual behaviors vary by context. For instance, in our study, selective allocation to MSM agents with greater than 10 expected partners in a year reached a maximum population coverage threshold of approximately 16%. The proportion of MSM engaging in high rates of partner turnover may be higher or lower in other settings. Nonetheless, this finding does suggest that too restrictive criteria may limit population coverage.

Nonetheless, our study corroborates prior findings that improvement in PrEP efficiency can be achieved by engaging those who meet current CDC criteria, though in our case, we did not model these criteria directly [11,12]. Given low rates of PrEP uptake nationally [9], our findings suggest that PrEP alone will be insufficient to produce a marked decrease in HIV incidence at population-level without significant increases in coverage. An analysis of the 2014 National HIV Behavioral Surveillance survey conducted in San Francisco, a city in which PrEP rollout has been more aggressive than in most areas, found that 9.9% of MSM (14.5% of those eligible) reported PrEP use within the last year [42]. These figures suggest substantial challenges lay ahead and that successful implementation of PrEP requires sustained focus on engaging populations at high risk of HIV infection.

### Limitations

Our study has a number of limitations. First, while our model takes into account various sources of behavioral and demographic heterogeneity, processes that plausibly drive observed disparities in Rhode Island among MSM are not accounted for. In many cases, a lack of data necessitates simplifying assumptions or omission of these processes. For instance, the model does not account for heterogeneities in sex frequency by age [43]; condom use patterns that differ by partner type (*eg*, main vs. casual) [20], HIV serostatus, and/or sexual role [20,21]; or age discordance [44,45]. However, we do vary the probability of condom use by the number of prior sexual episodes, which we opted for as an alternative to the treatment of partnerships as “main” or “casual”. Furthermore, in using monthly time steps, we were unable to explicitly account for brief partnerships (i.e., “one night stands”) and other short-term sexual behavior dynamics (e.g., group sex events). Because we focused on aggregate epidemiologic outputs over a 10-year simulation period, we do not believe the choice of time step would affect these measures substantially; however, using shorter time steps may facilitate a higher-resolution analysis of HIV transmission dynamics in this population.

Second, our model predicts rising incidence, though diagnoses in Rhode Island have remained relatively steady [13,46]. The trend in this model output is due possibly to our calibrating to a rising prevalence while imposing a steady mortality rate among HIV-infected agents. Surveillance data in Rhode Island suggests progression to AIDS and HIV/AIDS-related mortality is slowing, though these estimates are likely to be unstable [13,33]. Nonetheless, increasing prevalence brings with it the potential for a corresponding increase in incidence, which cannot be ruled out. The sex frequency sensitivity analyses allowed us to model PrEP in a population with substantially different background HIV incidence, indicating similar proportionate reductions in HIV incidence but with implications for PrEP efficiency.

Third, selection for PrEP did not take into account other indications for PrEP, such as recent condom use behavior prior to prescription. It is conceivable, therefore, that simulated measures of PrEP efficiency are slightly pessimistic in the allocation scenarios. However, while 8% of the MSM in the model were assigned an annual partner number mean of 10 or greater at model initialization (or upon the agent’s entry into the population), the PN > 10 PrEP allocation scenario achieved approximately 15–17% population coverage, due to the fact that agents with lower annual means stochastically drew a target partner number matching the scenario criterion in a given year, increasing the population of MSM indicated for PrEP. Given this information, the maximal impact for this scenario may in fact be lower than that observed in the model. Having modeled alternative levels of coverage, the 5% and 10% scenarios may be the most informative in predicting the likely effects of allocating PrEP to MSM with at least 10 partners annually.

Fourth, we did not model behavioral risk compensation, such as a decrease in condom use or an increase in partner acquisition rate, for agents initiating PrEP. An early study of MSM initiating PrEP in Rhode Island indicated a higher number of partnerships within which condomless anal sex occurred but not an overall increase in the number of partners over the first 6 months of follow-up [47]. We also did not implement the (approximately) 3-month HIV screening interval among MSM agents on PrEP, who continue in the model to test stochastically at the background rate.

Fifth, MSM from Rhode Island are likely to engage in partnerships with men from other states and, possibly, to seek PrEP from outside providers. We did not account for these potentialities. The model therefore may overestimate HIV incidence attributable to in-state sexual partnerships. Nonetheless, HIV incidence was calibrated based on new and prevalent diagnoses reported among Rhode Island residents, regardless of the source partner’s state of residence, and these are the infections we would seek to prevent. As new diagnoses are not necessarily incident infections, however, surveillance data cannot rule out the presence of imported infections [13]. MSM seeking PrEP from out-of-state sources would not be captured in the Rhode Island PrEP clinic data, which we acknowledge is an inherent limitation.

Sixth, in future studies using this model, we will attempt to employ more sophisticated calibration methods and sensitivity analyses. The sex frequency and partner number sensitivity analyses sought to examine how measures of PrEP impact and efficiency responded to fundamentally different patterns of transmission, but these analyses also featured markedly different dynamics than those underlying the main model.

Finally, a general challenge of modelling remains the considerable uncertainty surrounding model parameters. Different parameter sets could feasibly reproduce the incidence or prevalence trends sought, potentially affecting the underlying transmission dynamics. In our case, parameters governing sex frequency and condom use are derived from populations that may not necessarily resemble MSM in Rhode Island [20,23]. Recent research and commentary have attempted to understand the inferences drawn from ABMs within the counterfactual framework in causal inference [48–51]. We refer readers to two recent articles which elucidate the potential bias induced by erroneously assuming the portability of parameter estimates from external populations in the context of ABMs, a concern which may apply to this study [50,51]. We stress the importance of collecting detailed behavioral data in a range of settings, information which is indispensable for parameterizing agent-based models.

## Conclusions

This study sought to determine PrEP allocation strategies that maximize the population-level impact of a statewide PrEP implementation, with the aim of reaching the US national goal of reducing HIV transmission by 25% over 10 years. Most allocation scenarios achieved at least a 25% reduction in new infections when PrEP coverage was sustained at 15% of the HIV-negative population over 10 years. Focusing PrEP engagement for individuals with higher numbers of partners achieved the 25% reduction at lower coverage than other scenarios. New PrEP implementations at the state or city level should consider the importance of engaging MSM at especially high risk of HIV infection, both to increase the population-level impacts of PrEP and to reduce the number of prescriptions required to curb incidence.

## Supporting information

S1 Appendix Technical information. (DOCX)

S1 Table Age mixing sensitivity analysis. HIV prevalence and incidence at 0% PrEP coverage.
(DOCX)

S2 Table Age mixing sensitivity analysis. PrEP impact and efficiency at 15% coverage of HIV-negative MSM.
(DOCX)

S3 Table Ten-year summary statistics across PrEP allocation scenarios and coverage levels vs. base case (0% PrEP coverage). (DOCX)

S1 Fig Simulated model outputs vs. calibration targets. Dashed lines in error bars mark 95% simulation intervals, while solid lines depict the mean ± standard deviation (*bottom row*). Points represent calibration targets in all images. Y-axis scales set based on range of data.
(EPS)

S2 Fig Cumulative incidence over 10 years under allocation of PrEP to MSM under 45 years of age. Gray squares depict cumulative incidence within each age group in No PrEP scenario, and facet titles refer to PrEP coverage scenario. Point ranges encode medians and 95% simulation intervals.
(EPS)

S3 Fig Age-specific incidence rates per 1000 person-years at risk under differing mixing scenarios. Sensitivity analyses used the age mixing matrix 0%, 25%, or 75% of the time, and in all other instances resorted to random partner selection. The main analysis presented in our report utilized this matrix for 100% of partnerships. The dashed line encodes the 95% simulation interval, while the solid line encodes the mean plus/minus the standard deviation.
(EPS)

## Abbreviations

MSM: men who have sex with men
PrEP: pre-exposure prophylaxis
NHAS: National HIV/AIDS Strategy
NIA: number of infections averted
PIA: proportion of infections averted
PYPAI: person-years on PrEP per averted infection
