# Correction: Improving the impact of HIV pre-exposure prophylaxis implementation in small urban centers among men who have sex with men: An agent-based modelling study

**DOI:** 10.1371/journal.pone.0226218

**Published:** 2019-12-03

**Authors:** Jason R. Gantenberg, Maximilian King, Madeline C. Montgomery, Omar Galárraga, Mattia Prosperi, Philip A. Chan, Brandon D. L. Marshall

The following information is missing from the Funding statement: PAC received research funding and honoraria from Gilead Sciences within the five years prior to the conduct of the study.

The following information is missing from the Competing Interests statement: PAC received research funding and honoraria from Gilead Sciences within the five years prior to the conduct of the study.

## References

[1] Gantenberg JR, King M, Montgomery MC, Galárraga O, Prosperi M, Chan PA, et al (2018) Improving the impact of HIV pre-exposure prophylaxis implementation in small urban centers among men who have sex with men: An agent-based modelling study. PLoS ONE 13(7): e0199915 10.1371/journal.pone.0199915 29985949 PMC6037355

