# Supplementary material for: Improving the impact of HIV pre-exposure prophylaxis implementation in small urban centers among men who have sex with men: An agent-based modelling study
Source: PLoS One. 2018 Jul 9;13(7):e0199915. doi: 10.1371/journal.pone.0199915 (PMC6037355; doi:10.1371/journal.pone.0199915)
Supplement: S1 Appendix — (DOCX) [file pone.0199915.s001.docx]

S1 Appendix. Technical Information

Improving the impact of HIV pre-exposure prophylaxis implementation in small urban centers among men who have sex with men: An agent-based modelling study

Jason R. Gantenberg^1^, Maximilian King^1^, Madeline C. Montgomery^2^, Omar Galárraga^3^, Mattia Prosperi^4^, Philip A. Chan^2^, Brandon D.L. Marshall^1^

^1^ Department of Epidemiology, Brown University School of Public Health, Providence, RI, USA

^2^ Department of Medicine, Brown University, Providence, RI, USA

^3^ Department of Health Services, Policy, and Practice, Brown University School of Public Health, Providence, RI, USA

^4^ Department of Epidemiology, College of Public Health and Health Professions & College of Medicine, University of Florida, Gainesville, FL, USA

**Table of Contents**

[Abbreviations 2](#_Toc517454645)

[Note 2](#_Toc517454646)

[Purpose 2](#_Toc517454647)

[Model entities, characteristics, and scales 2](#_Toc517454648)

[Model overview 3](#_Toc517454649)

[Partnering 3](#_Toc517454650)

[Sexual activity 3](#_Toc517454651)

[HIV test 4](#_Toc517454652)

[PrEP start 4](#_Toc517454653)

[HIV/AIDS status 4](#_Toc517454654)

[Mortality and birth 4](#_Toc517454655)

[Observation 5](#_Toc517454656)

[Model design 5](#_Toc517454657)

[Adaptation 6](#_Toc517454658)

[Objectives 6](#_Toc517454659)

[Learning 6](#_Toc517454660)

[Sensing 6](#_Toc517454661)

[Interaction 6](#_Toc517454662)

[Stochasticity 6](#_Toc517454663)

[Initialization 7](#_Toc517454664)

[Overall population size 8](#_Toc517454665)

[Age distributions 8](#_Toc517454666)

[HIV prevalence disparities 8](#_Toc517454667)

[Demography 9](#_Toc517454668)

[Population 9](#_Toc517454669)

[Mortality 9](#_Toc517454670)

[Sexual networks and behavior 11](#_Toc517454671)

[Sexual relationships 11](#_Toc517454672)

[Condom use 11](#_Toc517454673)

[Sexual role 12](#_Toc517454674)

[Sex frequency 12](#_Toc517454675)

[Relationship duration 13](#_Toc517454676)

[Age mixing 14](#_Toc517454677)

[HIV/AIDS 15](#_Toc517454678)

[HIV transmission 16](#_Toc517454679)

[HIV treatment and disease 17](#_Toc517454680)

[AIDS 17](#_Toc517454681)

[Viral suppression 18](#_Toc517454682)

[PrEP scenarios 18](#_Toc517454683)

[The Miriam Hospital Clinic 19](#_Toc517454684)

[PrEP adherence and retention 19](#_Toc517454685)

[Characteristics of PrEP patients 20](#_Toc517454686)

[Model calibration 21](#_Toc517454687)

[Sensitivity analyses 22](#_Toc517454688)

[Technical details 25](#_Toc517454689)

[References 29](#_Toc517454690)

#

# Abbreviations

PrEP pre-exposure prophylaxis

ABM agent-based model

MSM men who have sex with men

NCHHSTP National Center for HIV/AIDS, Hepatitis, STD, and TB Prevention

CAS condomless anal sex

ART antiretroviral therapy

ADSR absolute difference in square roots

PLWH people living with HIV

# Note

While it is not implemented formally or in full, this supplement incorporates selected descriptive elements, terms, headings, and concepts from the ODD protocol [[1,2]](https://paperpile.com/c/d6r85Y/9i69Z+JfKqn).

# Purpose

In this study, we sought to examine the impacts of imposing simple demographic and behavioral targeting criteria for pre-exposure prophylaxis (PrEP) on population-level and within-group epidemiologic indicators of HIV transmission among men who have sex with men (MSM) in Rhode Island. Because the population-level effects of PrEP, including disparities in HIV burden, will depend in part on patterns of administration, we aimed to inform implementation efforts using criteria that are easily ascertained in the context of a clinical visit. PrEP efficiency has also been shown to vary based on underlying HIV incidence and targeting of PrEP prescriptions [[3–5]](https://paperpile.com/c/d6r85Y/SvkS5+hSx8A+FyKAF).

# Model entities, characteristics, and scales

The sole entities contained in the model are agents, who represent individual MSM. We do not incorporate spatial scales, and therefore, agents interact without respect to geographic proximity. MSM agents are characterized according to both demographic characteristics (age), sexual behavior (annual partner number, sex frequency, sexual position), and HIV status (positive or negative). HIV-negative agents are further characterized by their PrEP status (on/off, fully/partially adherent), while HIV-positive agents are characterized by their HIV testing status (diagnosed/aware or unaware) and by their antiretroviral treatment status (none; ART, non-adherent and not virally suppressed; ART, adherent and virally suppressed).

The model progresses in discrete time steps representing one month. After a 6-year burn-in period matching HIV prevalence between end-of-year 2008 and end-of-year 2014, the model is run for a further 10 years; in PrEP scenarios, the beginning of this 10-year window marks the first introduction of PrEP, as well as the analytic window of interest. Each scenario is simulated 1000 times.

# Model overview

## Partnering

During each time step, agents determine their need for a partner based on their target for the current year and, if indicated, seek out and pair with other searching agents. Based on the index agent’s sexual role class, a partner is selected from a list of other partner-seeking agents with a compatible sexual role such that exclusively receptive agents cannot pair with one another nor can exclusively insertive agents. With a probability governed by the age mixing matrix (see *Age Mixing*), the age group of origin for the partner is determined, and 100 partner-seeking agents from that group are enumerated. One, then, is selected at random.

This process is part of a negative binomial searching process in which partners are drawn from the population until the agent has achieved the necessary number of partners for the time step. The set of partner-seeking agents are then iterated through utilizing a *k*-nearest neighbor matching algorithm where subsets of the searching population are subdivided by suitable matches, and then stochastically assigned partners until the partner-seeking pool is exhausted. This process was implemented to reduce computational costs of the partnering algorithm while allowing flexibility to set matching constraints around sexual role and age. Once a match is found, a relationship edge is formed between the agent nodes, with a set duration drawn from the relationship duration distribution observed from Wall et al [[6]](https://paperpile.com/c/d6r85Y/ErmSP).

Each agent is assigned a distribution for their annual partner number mean, and each year, agents are assigned a new target partner number drawn from this distribution. Target partner numbers are updated in discrete 12-month intervals rather than on a rolling basis. In this way, an agent can be thought of as having particular proclivities with regard to partner acquisition but is not deterministically held to that behavior over time. Annual partner number means are distributed among the population based on STI clinic data from Rhode Island [[7]](https://paperpile.com/c/d6r85Y/8M2zL).

Upon formation, partnerships are assigned an expiration date based on relationship duration estimates published in the literature [[6]](https://paperpile.com/c/d6r85Y/ErmSP).

Agent dictionaries are updated to reflect new partner acquisitions at the completion of each index agent’s search.

## Sexual activity

Each agent has a desired number of sex acts per partner per year [[6]](https://paperpile.com/c/d6r85Y/ErmSP). At the partnership level, the number of monthly sex acts within a partnership represents a “compromise” between the paired agents’ preferences. The model simulates at-risk sexual episodes during each time step, defined as unprotected episodes within serodiscordant partnerships. The probability of condom use is determined by the sexual history between each pair of agents, namely the number of prior sexual contacts with that person [[8]](https://paperpile.com/c/d6r85Y/zVbWE).

Infection events occur stochastically according to a binomial process model in which the probability of infection per time step depends upon the number of unprotected acts, the PrEP status and sexual role of the HIV-negative partner, and the HIV-positive partner’s awareness of their own infection, in addition to their viral suppression status.

## HIV test

Once all agents have completed their sexual interactions for the current time step, agents seek HIV testing based on data from The Miriam Hospital STI Clinic [[7]](https://paperpile.com/c/d6r85Y/8M2zL). The annual testing rate was set at 62% of MSM each year and calibrated to match the intended proportion of diagnosed HIV-positive individuals. Initially, 89% of MSM were considered to have ever tested. No restriction on re-testing is imposed; in other words, an agent may test multiple times within a year.

## PrEP start

The number of available PrEP prescriptions is determined based on the desired population coverage, defined as the proportion of the HIV-negative population covered throughout the analytic simulation window. Because HIV transmission is stochastic, the size of the susceptible population may in fact change over time—for instance, as prevalence rises—but the number of prescriptions available remains steady. However, population coverage in most cases does not vary around the set target by more than 1–2%.

Agents initiate PrEP stochastically based on the active PrEP allocation scenario (see *PrEP Allocation Scenarios*). In all cases, adherence and dropout rates are based on data from the Miriam Clinic population, described in more detail in the section that discusses the Rhode Island PrEP program.

## HIV/AIDS status

HIV-positive agents can be virally suppressed or not, and they may be aware of their infection or not. Both viral suppression and awareness decrease the agent’s probability of transmitting to a susceptible partner. See the section entitled *HIV/AIDS* for more information regarding HIV transmission, treatment, and disease.

## Mortality and birth

The section entitled *Demography* contains information regarding underlying population processes. Briefly, the model contains agents between the ages of 15 and 74. Agents exit the model stochastically based on age-specific mortality rates and deterministically upon reaching the age of 75. Agents who exit the model through either process are replaced by a new HIV-negative agent from the same age group.

## Observation

At the end of each time step, the model records the total number of:

- Individuals in the population
- HIV-related deaths
- Total HIV-positive agents
- Diagnosed HIV-positive agents
- ART-adherent agents (i.e., virally suppressed)
- Agents on PrEP
- Agents newly infected with HIV
- Agents newly diagnosed with HIV

Breakouts by age are included for each of these measures.

# Model design

The primary outputs with regard to our study are the absolute and relative reductions in HIV incidence in addition to PrEP efficiency. Decreases in cumulative incidence are defined as the number of infections averted (NIA) and proportion of infections averted (PIA) and summarized by characterizing a distribution of cumulative incidence measures for each PrEP allocation scenario around the mean cumulative incidence of a scenario in which PrEP was never introduced. This method is based on that of Jenness et al [[3,9]](https://paperpile.com/c/d6r85Y/SvkS5+AfFvz).

Underlying these outputs, we have represented a simplified sexual activity and mixing structure which seeks to introduce a network topology determined by sexual position and age mixing. Furthermore, we have incorporated the effects of HIV testing, treatment, and pre-exposure prophylaxis, all of which reduce the risk of per-act transmission, either by explicitly representing treatment efficacy, in the case of ART or PrEP, or by implicitly assuming general reductions in the sexual risk behaviors among HIV-positive MSM aware of their infections.

During the burn-in period, the model attempts to recreate the observed increase in HIV prevalence among MSM in Rhode Island from the end of 2008 through 2014. In addition, it seeks to reproduce the proportionate disparities in HIV incidence by age (assessed independently). These proportionate incidences, in conjunction with overall HIV prevalence over time, represented our primary calibration targets.

In addition, the model is implemented in such a way as to maintain a steady proportion of HIV-positive MSM who are aware of their infection (i.e., are diagnosed) according to a Rhode Island estimate produced by Hall et al [[10]](https://paperpile.com/c/d6r85Y/GWT3h). This proportion arises out of the interaction between HIV transmission rates, mortality rates that differ by HIV and HIV treatment status, and the monthly testing probability for agents in the model. The final parameter is likely the most important in maintaining the desired levels of awareness.

## Adaptation

While not implemented as an explicit change to their behavioral patterns (e.g., no change in partnership acquisition rate or sexual frequency is imposed), HIV-positive agents pose less of a transmission risk to their HIV-negative partners. We use an assumed scaling factor of 0.5 on the per-act transmission risk from agents of this type, based on general observations that HIV-positive MSM aware of their infections are likely to reduce certain risk behaviors, such as condomless sex and partner acquisition, in the wake of a diagnosis [[11–15]](https://paperpile.com/c/d6r85Y/WW509+qHmnE+BxvdC+7SUK5+dwH7Z).

## Objectives

Based on their current annual partner number and the state of their current and past sexual partnerships, agents may in a particular time step have the objective of obtaining one or more partners. However, this objective represents a transient state and does not suggest that agents are modelled as having particular goals beyond the immediate acquisition of a partner. (See Grimm and Railsback for an explanation of *objectives* in the context of an individual-based simulation [[2]](https://paperpile.com/c/d6r85Y/JfKqn).)

## Learning

Agents do not learn or adapt over time, though they do engage in certain behaviors conditionally upon their partnership types and histories.

## Sensing

During the partnering process, individuals who inhabit an exclusive role preference (e.g., exclusively insertive, exclusively receptive) will not form partnerships with other agents within the same role preference class. Agents searching for partners will attempt to find the best fitting partners according to age (see *Age Mixing*). As mentioned previously, agents also engage in condomless anal sex (CAS) based on the number of prior sexual contacts, with the probability of condom usage decreasing as this measure of “familiarity” increases.

## Interaction

Agents interact in the model via sexual partnerships. Neither social networks nor agent-by-environment interactions are modelled, which is to say that agents do not alter their behavior based on system-level properties.

More specifically, agents may enter relationships with other agents and, within those partnerships, engage in condomless anal sex, and this represents the driving form of interaction during simulation. The probabilities that govern the interaction patterns and mixing structures are discussed in relevant sections later in this Appendix.

## Stochasticity

Stochasticity governs a number of processes in the model, summarized in the table below.

| **Parameter/Process** | **Purpose** |
| --- | --- |
| HIV transmission | Imposes variable infection risk by type of sexual exposure (insertive vs. receptive condomless anal sex). |
| Partner number | Allows for behavioral variability from year to year while maintaining some consistency in a given agent’s predisposition for partnership acquisition. |
| Assortative sexual mixing (by age) | Imposes baseline expected proportions for the distribution of partnerships in the population under random mixing, which are then tuned to introduce assortativity. |
| Relationship duration | Reproduces a discrete distribution of relationship durations. |
| Condom use probability | Imposes behavioral variability according to an agent’s familiarity with a given partner, based on the number of prior sexual contacts with that partner. |
| Sex frequency | Reproduces the discrete distribution of sexual frequency preferences in the population. The actual distribution represents a series of “compromises” between agents, in which individual sex frequency preferences are averaged within a partnership. |
| Viral suppression | Assigns a proportion of HIV-infected agents to be virally suppressed, with a 96% reduction in transmission risk to the uninfected partner. |
| PrEP initiation and dropout | Agents are selected for PrEP with a probability that changes based on the allocation scenario and drop out at random. |
| HIV testing | Testing occurs stochastically at a rate tuned to achieve the desired diagnosed proportion among HIV-infected agents. |
| Model initialization | Baseline demographic and behavioral characteristics in the seed population are all assigned stochastically. |

# Initialization

The model is initialized in a virtual population of 25,000 agents representing MSM aged 15 to 74 in Rhode Island. For each model run, the base population is seeded stochastically to generate the desired age distribution and age-specific prevalence of HIV among MSM, in addition to behavioral and HIV treatment-related parameters that are assigned to each individual.

## Overall population size

The overall MSM population size was estimated based on two estimates published for Rhode Island [[16,17]](https://paperpile.com/c/d6r85Y/0l04J+OblL8). First, using the American Community Survey 2011–2015 5-year estimate for the male population size in Rhode Island [[18]](https://paperpile.com/c/d6r85Y/Yvgbx), we subtracted those aged 75 and over or under 15 years of age. Applying the estimate from Grey et al that 6% of the adult male population in Rhode Island are MSM, we generated a population size estimate of 23,519. Next, using the estimates from Lieb et al, who differentiated the proportion of males who are MSM by race/ethnicity (white, black, Hispanic, other), we calculated a summary probability of being MSM for minority males in the state, weighted by the MSM population size accounted for by each race in these authors’ results. Lieb et al reported that 7% of white males were MSM in Rhode Island, and based on the procedure above, we calculated that 5.97% of minority males were the same [[17]](https://paperpile.com/c/d6r85Y/OblL8). Applying these proportions to the CDC WONDER data, we generated a total population size of 26,517. Using these two estimates we settled on the final population size of 25,000. The overall Rhode Island population changed by less than 1% between 2010 and 2017 [[19]](https://paperpile.com/c/d6r85Y/93uwg), and without more detailed information on the MSM population size, we held the population size steady throughout the course of simulation.

## Age distributions

This base population is seeded according to age distributions using 2011–2015 estimates in the CDC WONDER database, which rely upon July 1 bridged-race estimates from the U.S. Census [[20]](https://paperpile.com/c/d6r85Y/t61ty).

| **AGE GROUP** | **PROPORTION OF MSM** |
| --- | --- |
| **15–24** | 0.202 |
| **25–34** | 0.174 |
| **35–44** | 0.159 |
| **45–54** | 0.191 |
| **55+** | 0.273 |

###

While the overall population count was estimated taking race/ethnicity into account, we did not model race/ethnicity in the final analysis and present only those figures relevant to calculating the population size.

## HIV prevalence disparities

Disparities in HIV prevalence were imposed at model initialization by age. We used surveillance data from the National Center for HIV/AIDS, Viral Hepatitis, STD, and TB Prevention (NCHHSTP) Atlas summarizing HIV outcomes attributable to male-to-male sexual contact for Rhode Island between 2008 and 2014 [[21]](https://paperpile.com/c/d6r85Y/JCyxW), taking the averaged yearly proportions of HIV/AIDS diagnoses attributable to each age group. (Notably, these figures omit those in the joint transmission category describing male-to-male sexual contact *and* injection drug use.) Furthermore, rather than attempting to recreate the fluctuations in diagnoses by group over time, we elected to implement the average disparity, due to low and/or unstable counts from year-to-year.

###

| **AGE GROUP** | **PROPORTION OF PREVALENT HIV INFECTIONS** | **HIV PREVALENCE WITHIN GROUP** |
| --- | --- | --- |
| **15–24*** | 0.035 | 0.006 |
| **25–34** | 0.144 | 0.029 |
| **35–44** | 0.250 | 0.055 |
| **45–54** | 0.377 | 0.069 |
| **55+** | 0.194 | 0.025 |
| ***** *Uses 13–24 bin from NCHHSTP Atlas* | | |

Within-group HIV prevalences were calculated using Bayes theorem, assuming an HIV prevalence of 3.5% at time 0, corresponding to the inferred prevalence of HIV among MSM in Rhode Island in 2008. This prevalence assumed the model population *N* of 25,000. In the final model, these age-specific prevalences are implemented, but we present mainly aggregate outputs for reasons discussed in *Model Calibration*.

# Demography

## Population

The ABM simulates a population of virtual agents representing MSM between the ages of 15–74 in Rhode Island. Agents exit the model deterministically upon exceeding the age of 74 or stochastically according to mortality rates that vary by disease status and HIV treatment. Agents who die or exit are replaced by HIV-negative agents with age drawn from the initial parameterized age distribution.

## Mortality

Age-specific mortality rates were implemented by aggregating data from the years 2011–2015, based on CDC WONDER statistics for Rhode Island [[22]](https://paperpile.com/c/d6r85Y/m8jaN). Because the CDC WONDER population denominators use July 1 estimates for each year, they should represent reasonable person-time estimates, assuming linear changes in population throughout a given year [[23]](https://paperpile.com/c/d6r85Y/H9IRX).

| **AGE GROUP** | **AGE-SPECIFIC MORTALITY*** |
| --- | --- |
| **15–24** | 0.64 |
| **25–34** | 1.39 |
| **35–44** | 2.11 |
| **45–54** | 4.71 |
| **55+** | 13.82 |
| * *Per 1000 person-years* | |

Additionally, agents with HIV/AIDS but without viral suppression were subject to a targeted overall mortality rate ratio of 3.8, based in part on several calculations using Rhode Island surveillance data from the NCHHSTP Atlas [[21]](https://paperpile.com/c/d6r85Y/JCyxW), which investigated differing assumptions regarding mortality among MSM living with HIV/AIDS in the state, and the existing scientific literature. For instance, we compared lagged deaths against the inferred previous end-of-year prevalence (similar to one prior approach [[24]](https://paperpile.com/c/d6r85Y/bx5c)), in addition to a scenario in which we assumed all deaths occurred among the 55% of the HIV-positive population without viral suppression. Age-standardized mortality rate ratios ranged between 2.1 and 3.8, but due to low and/or unstable counts, we informally combined this information with outside literature to determine the HIV-related mortality rate ratio.

A recent study from British Columbia in Canada estimated age-standardized mortality rate ratios among HIV-positive individuals never having initiated ART relative to HIV-negative individuals and found that this ratio decreased from 6.79 to 3.97 between 2007 and 2012 [[25]](https://paperpile.com/c/d6r85Y/DDjjs). Among males, the mortality ratio decreased from 3.72 to 2.33 during the same timeframe, but no breakout was provided by ART use. We considered a ratio of 3.0 to be conservative, considering the estimates among those never on ART from the Eyawo et al study [[25]](https://paperpile.com/c/d6r85Y/DDjjs). Two other studies investigated mortality rates in the pre- and post-HAART era, corroborating the overall decrease in mortality and the increased proportion of non-AIDS-related deaths among HIV-infected individuals [[26,27]](https://paperpile.com/c/d6r85Y/D3BWY+CaMEa). However, these studies did not provide age-standardized mortality rate ratios summarizing the relative risk of mortality in HIV-infected individuals vs. HIV-uninfected individuals.

We assumed individuals with current stage 3 disease (AIDS) were subject to a mortality rate ratio of 10, relative to the general population, estimated by averaging age-adjusted mortality rate ratios among individuals with starting CD4 counts of less than 200 cells/mm^3^ in a cohort of HIV-infected individuals initiating ART [[28]](https://paperpile.com/c/d6r85Y/zT9Ov). While this estimate comes from Denmark, we sought an age-adjusted rate ratio, and the resulting relative excess mortality vs. HIV-infected individuals without viral suppression is within the range of estimates provided by prior research [[29]](https://paperpile.com/c/d6r85Y/Hro8l).

The overall target mortality rate ratio is a weighted average of the mortality rate ratios for the HIV-positive non-AIDS population and the population of HIV-infected agents with AIDS, the weights being the proportion of the HIV-positive population accounted for by each group. Notably, we did not adjust the weights to account for the virally suppressed population, which would have changed the mortality rate ratio from 3.8 to 4.4. Given the inherent uncertainty in the relative excess of mortality, this discrepancy is unlikely to contribute meaningfully to the model outputs of interest.

Virally suppressed HIV-positive agents are assumed to have the same underlying background mortality rates as the general population, based on studies that have shown either similar life expectancy or comparable mortality among HIV-infected individuals with good clinical outcomes and timely initiation of HAART [[30–32]](https://paperpile.com/c/d6r85Y/uAezB+MTLZS+GtORR).

# Sexual networks and behavior

## Sexual relationships

The annual partner number distribution for the general population is taken from a recent study describing a sample of MSM reporting to the Miriam Hospital STI Clinic in Providence, Rhode Island [[7]](https://paperpile.com/c/d6r85Y/8M2zL). Given that this parameter is an estimate from an STI clinic convenience sample, it is possible the overall rate of partnership acquisition in the general population of MSM is overestimated.

| **Annual Partner Number** | **Proportion** |
| --- | --- |
| 0 | 0.08 |
| 1 | 0.24 |
| 2 | 0.16 |
| 3–4 | 0.18 |
| 5–10 | 0.25 |
| 10+ | 0.08 |
| *Source:* [[7]](https://paperpile.com/c/d6r85Y/8M2zL) | |

## Condom use

We used data from a study published by Rosenberger et al, to determine the per-act probability of CAS conditional upon the number of prior sexual contacts between two agents [[8]](https://paperpile.com/c/d6r85Y/zVbWE). This probability varies based on the cumulative number of sexual encounters between two agents, with only unprotected acts being simulated.

| **NUMBER OF SEXUAL CONTACTS WITH PARTNER** | **PROBABILITY OF CAS** |
| --- | --- |
| 1st | 0.443 |
| 2nd | 0.481 |
| 3rd–10th | 0.514 |
| > 10 | 0.759 |
| *CAS, condomless anal sex* | |

## Sexual role

Agents are assigned a fixed sexual position/role preference and may be exclusively insertive, exclusively receptive, or versatile. Versatile agents may engage in partnerships across all sexual role classes, while exclusive agents of either type are prohibited from pairing with one another. Using data published by Van Tieu et al from the EXPLORE study, which showed that approximately 86% of all MSM in their sample reported some versatility, we recalculated overall role proportions within the subset of individuals reporting condomless anal sex [[33]](https://paperpile.com/c/d6r85Y/HvcFM).

###

| **SEXUAL ROLE** | **PROPORTION OF MSM** |
| --- | --- |
| Top (insertive) | 0.187 |
| Bottom (receptive) | 0.108 |
| Versatile | 0.705 |

###

## Sex frequency

Data on the frequency of sex acts comes from a study by Wall et al of young MSM recruited via the internet, which reported annualized sexual frequency estimates based on responses concerning the respondent's most recent sexual partner [[6]](https://paperpile.com/c/d6r85Y/ErmSP). Because each agent has a “desired” sex frequency per partner per year, the pairing of two agents results in a compromise, or averaging, of their target frequencies. The yearly desired sex frequency per partner was first translated into a monthly rate, and this monthly rate was then used as the mean of a Poisson distribution from which the number of sex acts between the agent pair was drawn each month. While an individual agent’s desired sex frequency was not modified based on their desired number of partners for the year, the process of averaging partners’ sex frequency targets should reduce the occurrence of highly implausible rates of sexual activity for given agent dyads.

###

| **ANNUAL SEXUAL FREQUENCY**  **(# OF ACTS)** | **PROPORTION OF MSM** |
| --- | --- |
| 1 | 0.019 |
| 2–5 | 0.082 |
| 6–11 | 0.063 |
| 12–23 | 0.072 |
| 24–35 | 0.299 |
| 36–51 | 0.200 |
| 52–155 | 0.124 |
| > 155 | 0.141 |
| *Source:* [[6]](https://paperpile.com/c/d6r85Y/ErmSP) | |

###

## Relationship duration

The parameters for relationships durations are taken from the same study as the sexual frequency parameters and were also the durations of MSM respondents’ most recent relationships [[6]](https://paperpile.com/c/d6r85Y/ErmSP). Within the model, partnerships are assigned a duration at the time of formation based on the discrete distribution below.

| **RELATIONSHIP DURATION (MONTHS)** | **PROPORTION** |
| --- | --- |
| < 1 | 0.323 |
| 1–6 | 0.262 |
| 7–12 | 0.116 |
| 13–24 | 0.121 |
| 25–36 | 0.060 |
| > 37 | 0.118 |
| *Source:* [[6]](https://paperpile.com/c/d6r85Y/ErmSP) | |

This data possibly results in a more frequent rate of partnership dissolution than would be observed in the Rhode Island population of MSM, but we did not have access to primary network data on the relevant population.

## Age mixing

Research has suggested that younger MSM are at higher risk of HIV acquisition with older partners due to differences in underlying HIV prevalence, and age discordance has been associated with HIV infection, especially among younger MSM [[34–37]](https://paperpile.com/c/d6r85Y/Gc4wl+ggFjz+EhDy7+DJBLv). Furthermore, the ability of disassortative age mixing to drive HIV acquisition in younger MSM, independent of differences in sexual partner acquisition rates, has been demonstrated [[38]](https://paperpile.com/c/d6r85Y/Wf9HF). Age discordance has also been associated with lower probability of condom usage [[39]](https://paperpile.com/c/d6r85Y/Ig62s). Sullivan et al reported that 28% of MSM in a sample from Atlanta, GA, reported a partner 10 years or older [[40]](https://paperpile.com/c/d6r85Y/penFl), while Glick et al, analyzing data from the Urban Men’s Health Study and a sample of Seattle MSM aged 15–24, found that 16.7% and 28%, respectively, reported a recent sex partner 10 years or older [[41]](https://paperpile.com/c/d6r85Y/faTLo). A modelling study conducted among MSM in Sydney, Australia, however, found that 30% of partnerships occurred within 5-year age groups, assuming a uniform age distribution [[42]](https://paperpile.com/c/d6r85Y/1PzjE), suggesting that age mixing among MSM is, on average, assortative.

Without primary data from Rhode Island, we began with the assumption that individuals had a 50% chance of partnering with someone in their own 10-year age group (20-year age group in the case of our 55+ age category) and calculated discordant-pairing probabilities using the inverse of the absolute difference in the square roots (ASDR) of midpoint ages. We then distributed these probabilities among the remaining 50% of partnership possibilities.

For instance, the probability of a 15–24 year-old index agent (*i*)—where *Index Agent* refers to that searching for a sexual partnership—selecting a partner (*j*) in the 45–54 age group is:

$$Pr(j=45-54 | i=15-24) =\frac{w}{\Sigma w_{i}} \times0.5, w = \frac{1}{|\sqrt{19.5} - \sqrt{39.5}|}$$

where *w* is calculated only for out-of-group partnerships and multiplying by 0.5 adjusts each probability based on the prespecified 50% probability of within-group partnership.

This approach is adapted from prior agent-based modelling studies [[5,43]](https://paperpile.com/c/d6r85Y/lbz6U+FyKAF), which used the ASDR in different forms based on a network modelling approach by Krivitsky et al [[44]](https://paperpile.com/c/d6r85Y/V2tLi). Kasaie et al note that this statistic has one particularly desirable property: the effect of the ASDR on partnering probability decreases with increasing age [[5]](https://paperpile.com/c/d6r85Y/FyKAF). This property holds true in our model but in somewhat coarser fashion, due to the binning of age groups. Furthermore, because the denominator in the equation above will vary by the index agent’s age group, the likelihood of a 15–24 year-old selecting a 45–54 year-old will be similar but not identical to the reciprocal scenario.

|  | **EGO AGE (INDEX AGENT)** | | | | |
| --- | --- | --- | --- | --- | --- |
| **PARTNER AGE** | **15–24** | **25–34** | **35–44** | **45–54** | **55+** |
| **15–24** | 0.500 | 0.156 | 0.074 | 0.057 | 0.062 |
| **25–34** | 0.226 | 0.500 | 0.162 | 0.093 | 0.086 |
| **35–44** | 0.123 | 0.185 | 0.500 | 0.199 | 0.128 |
| **45–54** | 0.088 | 0.099 | 0.184 | 0.500 | 0.224 |
| **55+** | 0.064 | 0.061 | 0.079 | 0.150 | 0.500 |

# HIV/AIDS

Infection in the model occurs only within serodiscordant partnerships and varies by a number of factors, including:

- Sexual position of the HIV-negative partner (in versatile-versatile partnerships, the probability of insertive vs. receptive positioning assumed to be 50/50)
- Diagnosed status of the HIV-positive partner
- Treatment and viral suppression in the HIV-positive partner
- PrEP status of the HIV-negative partner

Baseline per-act transmission risks for insertive and receptive CAS are based on a systematic review by Patel et al [[45]](https://paperpile.com/c/d6r85Y/h1Ryr). The scalar for per-act risk reduction within dyads involving a virally suppressed partner was derived from the same study, taking the approximate average of the risk reductions for both insertive and receptive sex, which were very similar [[45]](https://paperpile.com/c/d6r85Y/h1Ryr). HIV-positive individuals who are diagnosed have a 50% lower chance of transmitting, as mentioned previously. Finally, HIV-negative individuals on PrEP were subject to decreases in per-act transmission probability of 96% and 76% for fully and partially adherent agents, respectively. (See *Adherence and Retention* in the section regarding The Miriam Hospital Clinic.) These risk reductions are based on data measuring the efficacy of combination tenofovir-emtricitabine at 4 or more doses per week and 2–3 doses per week [[46]](https://paperpile.com/c/d6r85Y/0Wo9E).

## HIV transmission

| **MODIFIER** | **TRANSMISSION FACTOR** | **SOURCE** |
| --- | --- | --- |
| **Sexual Position of HIV-uninfected Partner** | *Baseline Per-Act Probability* |  |
| Insertive | 0.0011 | [[45]](https://paperpile.com/c/d6r85Y/h1Ryr) |
| Receptive | 0.0138 | [[45]](https://paperpile.com/c/d6r85Y/h1Ryr) |
|  |  |  |
| **HIV Status** | *Per-Act Risk Reduction* |  |
| On ART, virally suppressed | 0.96 | [[45]](https://paperpile.com/c/d6r85Y/h1Ryr) |
| Diagnosed | 0.50 | Assumed |
|  |  |  |
| **PrEP Status** | *Per-Act Risk Reduction* |  |
| Fully adherent | 0.96 | [[46]](https://paperpile.com/c/d6r85Y/0Wo9E) |
| Partially adherent | 0.76 | [[46]](https://paperpile.com/c/d6r85Y/0Wo9E) |
| *ART, antiretroviral therapy; PrEP, pre-exposure prophylaxis* | | |

## HIV treatment and disease

| **INDICATOR** | **PROPORTION/**  **PROBABILITY** | **SOURCE** |
| --- | --- | --- |
| **PLWH with diagnosed infection** | 0.82^a^ | [[10]](https://paperpile.com/c/d6r85Y/GWT3h) |
|  |  |  |
| **Annual probability of obtaining an HIV test** | 0.62^b^ | [[7]](https://paperpile.com/c/d6r85Y/8M2zL) |
|  |  |  |
| **Treatment** |  |  |
| Proportion of PLWH on ART | 0.55 | Calculated^c^ [[47]](https://paperpile.com/c/d6r85Y/TYidw) |
| Proportion of PLWH,  virally suppressed | 0.45 | [[48,49]](https://paperpile.com/c/d6r85Y/aRNI2+dK3uw) |
|  |  |  |
| **Proportion of PLWH currently with AIDS^d^** | 0.11 | Calculated [[50]](https://paperpile.com/c/d6r85Y/gAId7) |
| *^a^ Note that this estimate was considered to be unstable due to a low average number of yearly diagnoses in Rhode Island.*  *^b^ Starting probability, manually tuned during calibration*  *^c^ Using Hall et al, 2013, we assumed approximately 82% of individuals on ART are virally suppressed and calculated the proportion as 0.45 / 0.55. The Rhode Island care continuum indicates a larger number of individuals with viral load suppression than are retained in care, but about 83% of those listed as ‘Engaged in Care’ are virally suppressed.  ^d^ Used only to calculate a weighted mortality rate ratio for individuals with HIV/AIDS* | | |

## AIDS

The number of individuals currently with AIDS was estimated roughly based on a combination of assumptions and data from Rhode Island. First, Touzard et al reported that 26.5% of all HIV-infected patients engaged in care at the Miriam Hospital Clinic in Providence, RI in 2010 had a last measured CD4 count of less than 350 cells/μl [[50]](https://paperpile.com/c/d6r85Y/gAId7). Assuming uniform distribution of CD4 measurements between 0 and 350, we estimated that 15% of all patients had a CD4 count ≤ 200 cells/mm^3^. Using this proportion as a baseline, we estimated the overall proportion of PLWH currently with AIDS, taking into account the proportion of PLWH engaged in care and the size of the undiagnosed population, and arrived at 11% as our final estimate of HIV-positive MSM with stage 3 disease at any given time [[10,48,49,51]](https://paperpile.com/c/d6r85Y/GWT3h+SIrhH+dK3uw+aRNI2). Notably, this calculation assumes that diagnosed individuals with AIDS are in care, due to inherent limitations in the available data. This figure was not used directly in the model but as a weight for the HIV/AIDS-related mortality rate multiplier (see *Mortality*).

## Viral suppression

HIV-positive agents may initiate ART and achieve viral suppression. The proportion of HIV-infected agents with viral suppression is targeted to be 45%, based on a recent care continuum for the state of Rhode Island [[48,49]](https://paperpile.com/c/d6r85Y/aRNI2+dK3uw). In this report, 55% of positively diagnosed males were virally suppressed. Applying this proportion to the whole population of MSM, assuming an 82% diagnosis rate, we arrived at the 45% estimate for viral suppression among MSM living with HIV/AIDS. Most transmissions among males in Rhode Island occur among MSM [[49]](https://paperpile.com/c/d6r85Y/dK3uw), so we considered the overall estimate for males to be reasonable, considering the care continuum did not differentiate individuals living with HIV attributed to male-to-male sexual contact. This report notes that no discrepancies in viral suppression were detected by age, and so we do not represent such disparities in the model. The model achieves the 45% target by assigning HIV-infected MSM to ART, with an 82% probability of achieving viral suppression [[47]](https://paperpile.com/c/d6r85Y/TYidw).

# PrEP scenarios

In each PrEP allocation scenario, PrEP is administered to agents using differing eligibility criteria selected either based on behavioral risk factors or within demographic groups that have experienced disparate transmission rates in Rhode Island in recent years.

PrEP allocation strategies implemented are summarized in the table below.

| **SCENARIO** | **DESCRIPTION** |
| --- | --- |
| **No PrEP** | - Base case scenario in which the model is run for the 6-year burn-in period and the 10-year simulation window |
| **Current patient population** | - The PrEP program resembles the current implementation in Rhode Island - Agents are selected, in order of priority, based on their annual partner number (quintiles) and age - Distributions within the clinic mirror those summarized in *Characteristics of PrEP patients* - Among scenarios featuring PrEP administration, this scenario is the “real-world” baseline for comparison. |
| **Random allocation** | - All HIV-negative MSM are eligible and selected for PrEP independent of any behavioral or demographic characteristics. - This scenario is provided as a control scenario against which implementing any selective PrEP allocation can be compared. |
| **High-degree nodes** | - Agents are eligible for PrEP based on their target partner number for the year, which they draw anew in each 12-month interval and achieve probabilistically. - Two allocation criteria in separate simulations:   - 5 or more partners (PN > 5)   - 10 or more partners (PN > 10) |
| **Young MSM** | - Agents aged 15–44 are the only agents eligible for PrEP - *Not reported in the main paper* |

In each allocation scenario that preferentially admits subpopulations of MSM into PrEP treatment, population-level coverage is limited by the proportion of HIV-negative agents in the targeted group, a proportion that varies across simulations due to multiple sources of stochasticity. It is for this reason we did not impose finer allocation criteria.

A word is warranted regarding differences between the Current patient population (CPP) scenario and the High-degree node scenarios. The CPP scenario included PrEP selection based in part on the age distribution of the Rhode Island clinic population (see *Characteristics of PrEP patients*), which skewed younger than the general population of MSM. In contrast, the high-degree node scenarios allocate PrEP to agents according to the background age distribution of the total MSM population (see *Age distributions*). Because we were unable to fully recreate age-specific incidence, younger MSM agents in the model are at lower risk of new infection compared to the likely reality. It would be expected, therefore, that the CPP scenario may underestimate the effectiveness of enrolling MSM who resemble the clinical population, and may exaggerate the relative gains to impact produced by targeting MSM with higher partner numbers.

With regard to PrEP eligibility, agents draw their target partner number for each 12-month interval and are considered to be PrEP-eligible in a given month based on this value, re-drawing their target at the beginning of the next yearly interval. Agents achieve partner number targets probabilistically, however, and their actual behavior may not comport in all cases with the PrEP eligibility criteria as stated. Nonetheless, behavior year-to-year should be correlated, as each agent draws their target each year from a personalized distribution with a static mean.

# The Miriam Hospital Clinic

In 2013, the Miriam Hospital Clinic began offering PrEP in accordance with CDC guidelines [[52]](https://paperpile.com/c/d6r85Y/maCJh). Data regarding the early stages of PrEP implementation in Rhode Island through the Miriam Clinic Hospital have been published previously [[53,54]](https://paperpile.com/c/d6r85Y/QUPTL+4swQB).

## PrEP adherence and retention

Agents which successfully enroll in the PrEP program were categorized into one of two PrEP adherence categories, which resulted in varying impact of the treatment on per-act HIV transmission probabilities. Of newly initiated agents, 82% of these were assumed to be fully adherent (≥ 4 doses per week) and the rest partially adherent (2–3 doses per week). In reality, this assessment of treatment adherence among PrEP users is likely to be somewhat optimistic. For this reason, we conducted sensitivity analyses varying the adherence patterns among agents assigned to PrEP.

When determining the monthly probability of dropout, we used a static probability of discontinuing PrEP enrollment which matched 4-month follow-up activity of empirical clinic retention. The value utilized was a 54% retention at 6 months since first PrEP initiation, resulting in an overall monthly discontinuation probability of 15% per agent actively enrolled, using a binomial process [[54]](https://paperpile.com/c/d6r85Y/4swQB).

## Characteristics of PrEP patients

Using a dataset summarizing the histories of the first 241 MSM to initiate PrEP, we calculated the annual partner distribution for individuals in the clinic based on observed follow-up histories. Briefly, first-time patients were asked to report the number of sexual partners during the past 90 days in which the respondent inhabited an exclusively insertive role, an exclusively receptive role, or a versatile role, with respect to sexual positioning. During follow-up visits, patients were asked to report this same behavior since their last clinic visit. Using the overall follow-up time for each individual, including the 90 days reported upon during the baseline visit, we estimated maximum and minimum annual partner number estimates and averaged the two in order to produce the partner number quintiles that determined selection for PrEP in the model’s CDC Guidelines scenario. The maximum partner number estimate assumed that every partner reported by the respondent during follow-up represented a unique individual. The minimum estimate, on the other hand, took the largest number of partners reported by a given respondent in a single visit and assumed all other reported contacts were included in this one report. For instance, if a respondent reported 2, 1, and 8 partners during three respective visits, the minimum estimate assumed the respondent had a total of 8 partners during follow-up, while the maximum estimate assumed 11.

Missing partner number estimates for a given role were assumed to be 0. While all PrEP agents were receiving care in Rhode Island, a small number of agents indicated residence outside of Rhode Island. We retained 1 patient with missing information regarding state of residence and a further 8 who lived in the neighboring state of Massachusetts. Patients living in New York, New Hampshire, California, or Florida were omitted (*n =* 4).

| **Variable** | **Median [IQR] or %** |
| --- | --- |
| **Annual Partner Number*** | 8 [4–17] |
|  |  |
| **Age Group** |  |
| 15–24 | 0.199 |
| 25–34 | 0.415 |
| 35–44 | 0.170 |
| 45–54 | 0.162 |
| 55+ | 0.054 |
| ** Uses middle estimate, as described in text; omits 1 patient with completely missing data for sex partners by sexual position. The 20th, 40th, 60th, and 80th percentiles for this distribution are as follows: 3, 6, 12, 20, respectively.* | |

# Model calibration

The model was calibrated manually, and parameters considered to be uncertain or which were necessary to achieve the observed prevalence trajectory in Rhode Island were tuned. These parameters include testing frequency, all-cause mortality, assortative mixing, annual sexual frequency, and rate of ART uptake (monthly).

Our calibration targets included HIV prevalence, proportion of HIV-positive individuals diagnosed, proportion of HIV-positive individuals virally suppressed, and proportionate incidence by age (S1 Fig). True HIV prevalence was inferred within each year assuming that a stable proportion of 82% of HIV infections were diagnosed [[10]](https://paperpile.com/c/d6r85Y/GWT3h), and the calibration target incorporates this inference. The overall tested proportion in the population was achieved by tuning the monthly probability of acquiring an HIV test among HIV-negative MSM. The proportion of HIV-positive individuals virally suppressed is achieved by specifying a monthly probability of ART initiation and subsequent adherence (approximately 82%) such that 45% achieve viral suppression [[47,48]](https://paperpile.com/c/d6r85Y/TYidw+aRNI2).

**S1 Fig. Simulated model outputs vs. calibration targets.** Dashed lines in error bars mark 95% simulation intervals, while solid lines depict the mean ± standard deviation (*bottom row*). Points represent calibration targets in all images. Y-axis scales set based on range of data.

To calibrate to age-specific incidence, we introduced a mixing matrix governing the probability of forming sexual partnerships within and across age groups. This matrix was assumed, and in the absence of other data, we did not impose scaling factors to achieve these incidence rates.

We also attempted to calibrate to proportionate incidence by age group based on reported disparities in diagnoses observed between 2008 and 2014 in Rhode Island. We sought to recreate this pattern by implementing assortative mixing by age, as described above. Briefly, proportionate incidence and incidence rates by age group were compared under several assortative mixing schemes, which varied the proportion of proposed partnerships in which the matrix was used to determine the probability of partnership formation. The scheme that provided the closest fit was used in the main analysis, and several other schemes are presented as sensitivity analyses. As discussed in the next section, we were unable to recreate incidence patterns by age and elected to present only aggregate outputs in the main analysis for this reason. Ultimately, we resorted to what we considered a more conservative approach: implementing age-based assortative mixing and investigating the effect of this one process on age-specific HIV incidence. Results pertaining to age therefore should be treated with caution, including estimates of PrEP impact using age as a targeting criterion (S2 Fig).

**S2 Fig.** **Cumulative incidence over 10 years under allocation of PrEP to MSM under 45 years of age.** Gray squares depict cumulative incidence within each age group in No PrEP scenario, and facet titles refer to PrEP coverage scenario. Point ranges encode medians and 95% simulation intervals.

# Sensitivity analyses

We conducted several one-way sensitivity analyses to investigate the potential effects of our underlying assumptions on model outputs of interest. S1 Table presents ending HIV prevalence, cumulative incidence, and incidence rates across alternative age-mixing scenarios, to investigate the effects of these assumptions on underlying transmission dynamics. S2 Table, on the other hand, presents measures related to PrEP administration. All scenarios were run using the *Current Patient Population* allocation scheme at 15% population coverage. Results from the other sensitivity analyses are presented in the main paper.

**S1 Table. Age mixing sensitivity analysis. HIV prevalence and incidence at 0% PrEP coverage.**

| **Scenario** | **HIV** | **Cumulative Incidence** | **Incidence Rate** | **Incidence Change**  **(%)** |
| --- | --- | --- | --- | --- |
| Main | 7.4  (6.7, 8.1) | 826  (711, 955) | 3.51  (3.00, 4.08) | - |
| *Age Mixing* |  |  |  |  |
| Mix0.00 | 7.1  (6.3, 7.9) | 777  (659, 902) | 3.30  (2.79, 3.85) | -5.9 |
| Mix0.25 | 7.2  (6.4, 7.9) | 784  (665, 905) | 3.33  (2.81, 3.86) | -5.1 |
| Mix0.50 | 7.2  (6.5, 8.0) | 797  (669, 929) | 3.38  (2.83, 3.96) | -3.5 |
| Mix0.75 | 7.3  (6.6, 8.1) | 815  (701, 928) | 3.47  (2.96, 3.96) | -1.3 |
| **Notes:**  Main, base case from main analysis  SF[*X*], sex frequency scale  PN[X], partner number scale  Mix[*X*], proportion of partner selections that use the age mixing matrix to determine the probability of partnership formation  *HIV*, ending HIV prevalence; *CumInc*, new infections over 10 years; *IR*, incidence rate per 1000 person-years at risk  Incidence change = percent change in 10-year median cumulative incidence relative to Main scenario  *Medians and 95% simulation limits reported* | | | | |

**S2 Table. Age mixing sensitivity analysis. PrEP impact and efficiency at 15% coverage of HIV-negative MSM.**

| **Scenario** | **NIA** | **PIA** | **PYPAI** |
| --- | --- | --- | --- |
| Main | 218  (121, 307) | 26.2  (14.5, 37.0) | 161  (115, 289) |
| *Age Mixing* |  |  |  |
| Mix0.00 | 202  (106, 294) | 25.9  (13.7, 37.9) | 174  (120, 329) |
| Mix0.25 | 203  (107, 292) | 25.9  (13.6, 37.2) | 174  (121, 327) |
| Mix0.50 | 207  (114, 296) | 26.0  (14.3, 37.2) | 170  (119, 306) |
| Mix0.75 | 211  (115, 305) | 25.9  (14.1, 37.5) | 167  (116, 304) |
| **Notes:**  *Main*, base case from main analysis  *Mix[X]*, proportion of partner selections using the age mixing matrix  *PrEP*, pre-exposure prophylaxis; *MSM*, men who have sex with men; *NIA*, number of infections averted, *PIA*, percentage of infections averted; *PYPAI*, person-years per averted infection  *Medians and 95% simulation limits reported* | | | |

Aggregate HIV transmission parameters were sensitive to sex frequency but not to the age mixing strategy used. Age-specific incidence measures were sensitive to the age mixing patterns (S3 Fig), as expected. Use of the age mixing matrix better reproduced the pattern of transmission across age groups but was apparently unable to overcome the HIV-related disparities imposed at baseline, in which older MSM agents were at considerably higher risk of prevalent infection. The most pronounced changes in incidence occurred within the two age groups covering 35–54 year-olds in the model, while incidence at the tails of the age distribution continued to deviate markedly from the specified targets. As discussed in our limitations, this result may be expected, considering assortative mixing by age is unlikely to explain by itself the observed age-related disparities in Rhode Island and elsewhere. Detailed primary network and treatment data specific to our population would be necessary to more closely reflect these determinants and patterns of HIV transmission, but for the most part, pertinent data were lacking.

**S3 Fig.** **Age-specific incidence rates per 1000 person-years at risk under differing mixing scenarios.** Sensitivity analyses used the age mixing matrix 0%, 25%, or 75% of the time, and in all other instances resorted to random partner selection. The main analysis presented in our report utilized this matrix for 100% of partnerships. The dashed line encodes the 95% simulation interval while the solid line encodes the mean plus/minus the standard deviation.

Figure 3 shows the NIA and PYPAI estimates across the range of PrEP adherence analyses, in which we altered the proportion of fully vs. partially adherent active PrEP users. PrEP measures did not appear to be sensitive to these assumptions. However, this analysis may yet underestimate the uncertainty regarding the adherence within the PrEP clinic. For instance, we do not model non-adherence. Rather, any agent on PrEP is subject either to a 96% or 76% reduction in HIV transmission risk. Ideally, PrEP patients should be followed up quarterly [[52]](https://paperpile.com/c/d6r85Y/maCJh), but it is possible that PrEP patients may use their prescription less frequently yet maintain therapeutic drug levels beyond their follow-up window, as long as they take at least 2 pills per week. (Note that we are by no means recommending this practice, given the lower efficacy associated with 2–4 doses/week [[46]](https://paperpile.com/c/d6r85Y/0Wo9E). We are simply discussing a factor that informed our choice of follow-up window.) If our assumptions were optimistic, we may be overestimating the effectiveness of PrEP in this lower-incidence setting when compared to other urban MSM populations.

Figure 4 depicts the effects of PrEP within settings of lower and higher incidence, achieved by scaling agents’ desired sex frequencies. Proportionate reductions in incidence are quite similar across these scenarios, but PrEP tends to be considerably more efficient when background incidence is higher.

# Technical details

Python™ (Version 2.7.13), an open-source programming language, was used for coding, testing, and calibrating the model. The simulations were run on Oscar, Brown University’s research computing cluster, which operates on the CentOS 6.7 Linux operating system and utilizes the SLURM workload manager. The simulations were processed using 2.53 GHz Intel Xeon E5540 processors operating with 8 cores at 14.84 Teraflops and 12GB of DDR3 memory. The model was run for a duration of 120 time-steps (10 years) and averaged over a total of 1000 Monte Carlo runs per scenario, each with a stochastically generated population following the parameters provided above. These 1000 runs were run in parallel and aggregated from bundles of 100-run units, with each unit having an average runtime of 2.36 hours.

Model output analysis and visualization was conducted in R 3.4 using a series of packages [[55–64]](https://paperpile.com/c/d6r85Y/M1p1K+06xuo+8VhM0+HD9DB+T5PzD+ThsJq+qtkMv+mWUDJ+Gst32+r1ugz).

**S3 Table. Ten-year summary statistics across PrEP allocation scenarios and coverage levels vs. base case (0% PrEP coverage).**

| **Scenario** | **HIV Prevalence (%)** | **New Infections** | **Incidence Rate*** | **Infections Averted (#)** | **Infections Averted (%)** | **PYPAI** |
| --- | --- | --- | --- | --- | --- | --- |
| *Coverage 0%* | | | | | | |
| No PrEP | 7.4  (6.7, 8.1) | 826  (711, 955) | 3.51  (3.00, 4.08) | - | - | - |
| *Coverage 5%* | | | | | | |
| Current patients | 7.1  (6.4, 7.9) | 754  (639, 874) | 3.20  (2.70, 3.73) | 76  (-44, 191) | 9.1  (-5.3, 23.0) | 154  (61, 11644) |
| Random | 7.1  (6.4, 7.9) | 768  (661, 890) | 3.26  (2.79, 3.80) | 62  (-60, 169) | 7.4  (-7.3, 20.3) | 189  (69, 11653) |
| PN > 5 | 7.0  (6.4, 7.8) | 741  (636, 860) | 3.14  (2.69, 3.66) | 89  (-30, 194) | 10.7  (-3.7, 23.3) | 132  (61, 11634) |
| PN > 10 | 7.0  (6.3, 7.7) | 731  (625, 842) | 3.10  (2.64, 3.58) | 99  (-12, 205) | 11.9  (-1.5, 24.7) | 118  (57, 11616) |
| *Coverage 10%* | | | | | | |
| Current patients | 6.8  (6.1, 7.5) | 681  (572, 786) | 2.89  (2.41, 3.35) | 149  (44, 258) | 17.9  (5.3, 31.1) | 158  (91, 535) |
| Random | 6.9  (6.2, 7.6) | 709  (601, 819) | 3.01  (2.53, 3.49) | 121  (11, 229) | 14.5  (1.3, 27.6) | 194  (103, 2198) |
| PN > 5 | 6.7  (6.1, 7.5) | 664  (571, 778) | 2.82  (2.41, 3.31) | 166  (52, 259) | 20.0  (6.2, 31.2) | 141  (91, 451) |
| PN > 10 | 6.6  (6.0, 7.3) | 635  (545, 739) | 2.69  (2.30, 3.14) | 195  (91, 285) | 23.5  (10.9, 34.3) | 120  (82, 257) |
| *Coverage 15%* | | | | | | |
| Current patients | 6.5  (5.9, 7.2) | 612  (523, 709) | 2.59  (2.21, 3.02) | 218  (121, 307) | 26.2  (14.5, 37.0) | 161  (115, 289) |
| Random | 6.7  (6.0, 7.4) | 654  (546, 756) | 2.77  (2.31, 3.22) | 176  (74, 284) | 21.2  (8.9, 34.2) | 199  (124, 474) |
| PN > 5 | 6.5  (5.8, 7.1) | 595  (499, 691) | 2.52  (2.11, 2.94) | 235  (139, 331) | 28.3  (16.7, 39.9) | 150  (107, 252) |
| PN > 10 | 6.3  (5.7, 6.9) | 555  (478, 639) | 2.35  (2.01, 2.71) | 275  (191, 352) | 33.1  (23.0, 42.4) | 128  (100, 184) |
| *Coverage 20%* | | | | | | |
| Current patients | 6.3  (5.7, 7.0) | 550  (468, 645) | 2.33  (1.98, 2.74) | 280  (185, 362) | 33.7  (22.3, 43.6) | 168  (130, 252) |
| Random | 6.5  (5.8, 7.2) | 602  (507, 699) | 2.55  (2.14, 2.97) | 228  (131, 323) | 27.4  (15.8, 38.9) | 206  (145, 356) |
| PN > 5 | 6.2  (5.6, 6.8) | 530  (450, 612) | 2.24  (1.90, 2.60) | 300  (218, 380) | 36.1  (26.2, 45.8) | 157  (124, 215) |
| PN > 10 | 6.3  (5.7, 6.9) | 540  (462, 626) | 2.28  (1.95, 2.66) | 290  (204, 368) | 34.9  (24.6, 44.3) | 130  (102, 183) |
| *Coverage 25%* | | | | | | |
| Current patients | 6.0  (5.5, 6.6) | 488  (412, 567) | 2.06  (1.73, 2.41) | 341  (263, 418) | 41.1  (31.7, 50.3) | 172  (141, 222) |
| Random | 6.3  (5.7, 7.0) | 553  (470, 652) | 2.34  (1.98, 2.77) | 277  (178, 360) | 33.3  (21.4, 43.4) | 211  (163, 328) |
| PN > 5 | 6.0  (5.4, 6.5) | 470  (398, 548) | 1.98  (1.68, 2.32) | 360  (282, 432) | 43.4  (33.9, 52.0) | 163  (136, 208) |
| PN > 10 | 6.2  (5.7, 6.9) | 537  (457, 633) | 2.27  (1.93, 2.69) | 293  (197, 373) | 35.3  (23.7, 44.9) | 129  (101, 190) |
| *Coverage 30%* | | | | | | |
| Current patients | 5.8  (5.3, 6.4) | 434  (370, 505) | 1.83  (1.56, 2.14) | 395  (325, 460) | 47.6  (39.1, 55.4) | 178  (154, 216) |
| Random | 6.1  (5.5, 6.8) | 503  (427, 589) | 2.13  (1.80, 2.50) | 327  (241, 403) | 39.4  (29.0, 48.5) | 216  (175, 290) |
| PN > 5 | 5.7  (5.2, 6.3) | 408  (345, 472) | 1.72  (1.45, 2.00) | 422  (358, 485) | 50.8  (43.1, 58.4) | 167  (146, 196) |
| PN > 10 | 6.3  (5.6, 6.9) | 540  (461, 631) | 2.29  (1.94, 2.68) | 290  (199, 369) | 34.9  (23.9, 44.4) | 131  (102, 188) |
| **Notes:** *HIV Prevalence*, ending HIV prevalence; *PYPAI*, person-years on PrEP per averted infection; *Current patients*, Current Patient Population scenario; *PN > 5*, expected annual partner number greater than 5; *PN > 10*, expected annual partner number greater than 10  Medians and 95% simulation limits presented. For simulation runs in which a PrEP scenario produced more infections than mean 10-year cumulative HIV incidence at 0% coverage, PYPAI set to the number of person-years on PrEP.  * Incidence rate per 1000 person-years at risk. | | | | | | |

# References

1. [Grimm V, Berger U, Bastiansen F, Eliassen S, Ginot V, Giske J, et al. A standard protocol for describing individual-based and agent-based models. Ecol Modell. 2006;198: 115–126.](http://paperpile.com/b/d6r85Y/9i69Z)

2. [Grimm V, Berger U, DeAngelis DL, Polhill JG, Giske J, Railsback SF. The ODD protocol: A review and first update. Ecol Modell. Elsevier; 2010;221: 2760–2768.](http://paperpile.com/b/d6r85Y/JfKqn)

3. [Jenness SM, Goodreau SM, Rosenberg E, Beylerian EN, Hoover KW, Smith DK, et al. Impact of the Centers for Disease Control’s HIV Pre-Exposure Prophylaxis Guidelines for Men Who Have Sex with Men in the United States. J Infect Dis. 2016; jiw223.](http://paperpile.com/b/d6r85Y/SvkS5)

4. [Carnegie NB, Goodreau SM, Liu A, Vittinghoff E, Sanchez J, Lama JR, et al. Targeting Pre-Exposure Prophylaxis Among Men Who Have Sex With Men in the United States and Peru: Partnership Types, Contact Rates, and Sexual Role. Journal of Acquired Immune Deficiency Syndromes. 05/2015;69: 119–125.](http://paperpile.com/b/d6r85Y/hSx8A)

5. [Kasaie P, Pennington J, Shah MS, Berry SA, German D, Flynn CP, et al. The Impact of Pre-Exposure Prophylaxis Among Men Who Have Sex With Men: An Individual-Based Model. J Acquir Immune Defic Syndr. 2017;75: 175–183.](http://paperpile.com/b/d6r85Y/FyKAF)

6. [Wall KM, Stephenson R, Sullivan PS. Frequency of Sexual Activity With Most Recent Male Partner Among Young, Internet-Using Men Who Have Sex With Men in the United States. J Homosex. 10/2013;60: 1520–1538.](http://paperpile.com/b/d6r85Y/ErmSP)

7. [Chan PA, Rose J, Maher J, Benben S, Pfeiffer K, Almonte A, et al. A Latent Class Analysis of Risk Factors for Acquiring HIV Among Men Who Have Sex with Men: Implications for Implementing Pre-Exposure Prophylaxis Programs. AIDS Patient Care STDS. 11/2015;29: 597–605.](http://paperpile.com/b/d6r85Y/8M2zL)

8. [Rosenberger JG, Reece M, Schick V, Herbenick D, Novak DS, Van Der Pol B, et al. Condom Use during Most Recent Anal Intercourse Event among a U.S. Sample of Men Who Have Sex with Men. J Sex Med. 2012;9: 1037–1047.](http://paperpile.com/b/d6r85Y/zVbWE)

9. [Jenness SM. PrEPGuidelines [Internet]. Github; Available:](http://paperpile.com/b/d6r85Y/AfFvz) <https://github.com/statnet/PrEPGuidelines>

10. [Hall HI, An Q, Tang T, Song R, Chen M, Green T, et al. Prevalence of Diagnosed and Undiagnosed HIV Infection--United States, 2008-2012. MMWR Morb Mortal Wkly Rep. 2015;64: 657–662.](http://paperpile.com/b/d6r85Y/GWT3h)

11. [Gorbach PM, Weiss RE, Jeffries R, Javanbakht M, Drumright LN, Daar ES, et al. Behaviors of recently HIV-infected men who have sex with men in the year postdiagnosis: effects of drug use and partner types. J Acquir Immune Defic Syndr. 2011;56: 176–182.](http://paperpile.com/b/d6r85Y/WW509)

12. [Khanna AS, Goodreau SM, Gorbach PM, Daar E, Little SJ. Modeling the Impact of Post-Diagnosis Behavior Change on HIV Prevalence in Southern California Men Who Have Sex with Men (MSM). AIDS Behav. 2013;18: 1523–1531.](http://paperpile.com/b/d6r85Y/qHmnE)

13. [Gorbach PM, Drumright LN, Daar ES, Little SJ. Transmission behaviors of recently HIV-infected men who have sex with men. J Acquir Immune Defic Syndr. 2006;42: 80–85.](http://paperpile.com/b/d6r85Y/BxvdC)

14. [Marks G, Crepaz N, Senterfitt JW, Janssen RS. Meta-analysis of high-risk sexual behavior in persons aware and unaware they are infected with HIV in the United States: implications for HIV prevention programs. J Acquir Immune Defic Syndr. 2005;39: 446–453.](http://paperpile.com/b/d6r85Y/7SUK5)

15. [Colfax GN, Buchbinder SP, Cornelisse PGA, Vittinghoff E, Mayer K, Celum C. Sexual risk behaviors and implications for secondary HIV transmission during and after HIV seroconversion. AIDS. 2002;16: 1529–1535.](http://paperpile.com/b/d6r85Y/dwH7Z)

16. [Grey JA, Bernstein KT, Sullivan PS, Purcell DW, Chesson HW, Gift TL, et al. Estimating the Population Sizes of Men Who Have Sex With Men in US States and Counties Using Data From the American Community Survey. JMIR Public Health and Surveillance. 2016;2: e14.](http://paperpile.com/b/d6r85Y/0l04J)

17. [Lieb S, Fallon SJ, Friedman SR, Thompson DR, Gates GJ, Liberti TM, et al. Statewide Estimation of Racial/Ethnic Populations of Men Who Have Sex with Men in the U.S. Public Health Rep. 2011;126: 60–72.](http://paperpile.com/b/d6r85Y/OblL8)

18. [US Census Bureau. 2011-2015 American Community Survey 5-Year Estimates. 2016.](http://paperpile.com/b/d6r85Y/Yvgbx)

19. [U.S. Census Bureau QuickFacts selected: Rhode Island. In: United States Census Bureau [Internet]. [cited 21 Oct 2017]. Available:](http://paperpile.com/b/d6r85Y/93uwg) <https://www.census.gov/quickfacts/RI>

20. [United States Department of Health and Human Services (US DHHS), Centers for Disease Control and Prevention (CDC), National Center for Health Statistics (NCHS). Bridged-Race Population Estimates, United States July 1st resident population by state, county, age, sex, bridged-race, and Hispanic origin [Internet]. Available:](http://paperpile.com/b/d6r85Y/t61ty) <https://wonder.cdc.gov/controller/saved/D134/D17F709>

21. [Atlas Plus | National Center for HIV/AIDS, Hepatitis, STD, and TB Prevention | Centers for Disease Control and Prevention [Internet]. [cited 13 Jul 2017]. Available:](http://paperpile.com/b/d6r85Y/JCyxW) <https://www.cdc.gov/nchhstp/atlas/>

22. [Centers for Disease Control and Prevention, National Center for Health Statistics. Underlying Cause of Death 1999-2015 on CDC WONDER Online Database, released December, 2016. Data are from the Multiple Cause of Death Files, 1999-2015, as compiled from data provided by the 57 vital statistics jurisdictions through the Vital Statistics Cooperative Program [Internet]. [cited 19 Jul 2017]. Available:](http://paperpile.com/b/d6r85Y/m8jaN) <https://wonder.cdc.gov/controller/saved/D76/D17F932>

23. [Vandenbroucke JP, Pearce N. Incidence rates in dynamic populations. Int J Epidemiol. 2012;41: 1472–1479.](http://paperpile.com/b/d6r85Y/H9IRX)

24. [An Q, Song R, Hernandez A, Hall HI. Trends and Differences Among Three New Indicators of HIV Infection Progression. Public Health Rep. 2015;130: 468–474.](http://paperpile.com/b/d6r85Y/bx5c)

25. [Eyawo O, Franco-Villalobos C, Hull MW, Nohpal A, Samji H, Sereda P, et al. Changes in mortality rates and causes of death in a population-based cohort of persons living with and without HIV from 1996 to 2012. BMC Infect Dis. 2017;17: 174.](http://paperpile.com/b/d6r85Y/DDjjs)

26. [Wada N, Jacobson LP, Cohen M, French A, Phair J, Muñoz A. Cause-specific mortality among HIV-infected individuals, by CD4(+) cell count at HAART initiation, compared with HIV-uninfected individuals. AIDS. 2014;28: 257–265.](http://paperpile.com/b/d6r85Y/D3BWY)

27. [Wada N, Jacobson LP, Cohen M, French A, Phair J, Muñoz A. Cause-Specific Life Expectancies After 35 Years of Age for Human Immunodeficiency Syndrome-Infected and Human Immunodeficiency Syndrome-Negative Individuals Followed Simultaneously in Long-term Cohort Studies, 1984–2008. Am J Epidemiol. 2013;177: 116–125.](http://paperpile.com/b/d6r85Y/CaMEa)

28. [Jensen-Fangel S, Pedersen L, Pedersen C, Larsen CS, Tauris P, Møller A, et al. Low mortality in HIV-infected patients starting highly active antiretroviral therapy: a comparison with the general population. AIDS. 2004;18: 89–97.](http://paperpile.com/b/d6r85Y/zT9Ov)

29. [Moore RD, Chaisson RE. Natural history of HIV infection in the_era of combination antiretroviral therapy. AIDS. 1999;13: 1933.](http://paperpile.com/b/d6r85Y/Hro8l)

30. [Samji H, Cescon A, Hogg RS, Modur SP, Althoff KN, Buchacz K, et al. Closing the Gap: Increases in Life Expectancy among Treated HIV-Positive Individuals in the United States and Canada. PLoS One. 2013;8: e81355.](http://paperpile.com/b/d6r85Y/uAezB)

31. [Rodger AJ, Lodwick R, Schechter M, Deeks S, Amin J, Gilson R, et al. Mortality in well controlled HIV in the continuous antiretroviral therapy arms of the SMART and ESPRIT trials compared with the general population. AIDS. journals.lww.com; 2013;27: 973–979.](http://paperpile.com/b/d6r85Y/MTLZS)

32. [Lewden C, Chêne G, Morlat P, Raffi F, Dupon M, Dellamonica P, et al. HIV-Infected Adults With a CD4 Cell Count Greater Than 500 Cells/mm3 on Long-Term Combination Antiretroviral Therapy Reach Same Mortality Rates as the General Population. Journal of Acquired Immune Deficiency Syndromes. 2007;46: 72–77.](http://paperpile.com/b/d6r85Y/GtORR)

33. [Tieu H-V, Li X, Donnell D, Vittinghoff E, Buchbinder S, Parente ZG, et al. Anal sex role segregation and versatility among men who have sex with men: EXPLORE Study. J Acquir Immune Defic Syndr. 2013;64: 121–125.](http://paperpile.com/b/d6r85Y/HvcFM)

34. [Hurt CB, Matthews DD, Calabria MS, Green KA, Adimora AA, Golin CE, et al. Sex with older partners is associated with primary HIV infection among men who have sex with men in North Carolina. J Acquir Immune Defic Syndr. 2010;54: 185–190.](http://paperpile.com/b/d6r85Y/Gc4wl)

35. [Anema A, Marshall BDL, Stevenson B, Gurm J, Montaner G, Small W, et al. Intergenerational sex as a risk factor for HIV among young men who have sex with men: a scoping review. Curr HIV/AIDS Rep. 2013;10: 398–407.](http://paperpile.com/b/d6r85Y/ggFjz)

36. [Berry M, Raymond HF, McFarland W. Same race and older partner selection may explain higher HIV prevalence among black men who have sex with men. AIDS. 2007;21: 2349–2350.](http://paperpile.com/b/d6r85Y/EhDy7)

37. [Chamberlain N, Mena LA, Geter A, Crosby RA. Is Sex with Older Male Partners Associated with Higher Sexual Risk Behavior Among Young Black MSM? AIDS Behav. 2017; doi:](http://paperpile.com/b/d6r85Y/DJBLv)[10.1007/s10461-017-1699-4](http://dx.doi.org/10.1007/s10461-017-1699-4)

38. [Service SK, Blower SM. HIV transmission in sexual networks: an empirical analysis. Proceedings of the Royal Society of London B: Biological Sciences. The Royal Society; 1995;260: 237–244.](http://paperpile.com/b/d6r85Y/Wf9HF)

39. [Del Pino HE, Harawa NT, Liao D, Moore AA, Karlamangla AS. Age and Age Discordance Associations with Condomless Sex Among Men Who Have Sex with Men. AIDS Behav. 2017; doi:](http://paperpile.com/b/d6r85Y/Ig62s)[10.1007/s10461-017-1694-9](http://dx.doi.org/10.1007/s10461-017-1694-9)

40. [Sullivan PS, Rosenberg ES, Sanchez TH, Kelley CF, Luisi N, Cooper HL, et al. Explaining racial disparities in HIV incidence in black and white men who have sex with men in Atlanta, GA: a prospective observational cohort study. Ann Epidemiol. 2015;25: 445–454.](http://paperpile.com/b/d6r85Y/penFl)

41. [Glick SN, Morris M, Foxman B, Aral SO, Manhart LE, Holmes KK, et al. A Comparison of Sexual Behavior Patterns Among Men Who Have Sex With Men and Heterosexual Men and Women. Journal of Acquired Immune Deficiency Syndromes. 05/2012;60: 83–90.](http://paperpile.com/b/d6r85Y/faTLo)

42. [Wilson DP. Modelling based on Australian HIV notifications data suggests homosexual age mixing is primarily assortative. Journal of Acquired Immune Deficiency Syndromes. 2009;51: 356–360.](http://paperpile.com/b/d6r85Y/1PzjE)

43. [Goodreau SM, Carnegie NB, Vittinghoff E, Lama JR, Sanchez J, Grinsztejn B, et al. What Drives the US and Peruvian HIV Epidemics in Men Who Have Sex with Men (MSM)? PLoS One. 2012;7: e50522.](http://paperpile.com/b/d6r85Y/lbz6U)

44. [Krivitsky PN, Handcock MS, Morris M. Adjusting for network size and composition effects in exponential-family random graph models. Stat Methodol. 2011;8: 319–339.](http://paperpile.com/b/d6r85Y/V2tLi)

45. [Patel P, Borkowf CB, Brooks JT, Lasry A, Lansky A, Mermin J. Estimating per-act HIV transmission risk: a systematic review. AIDS. 06/2014;28: 1509–1519.](http://paperpile.com/b/d6r85Y/h1Ryr)

46. [Anderson PL, Glidden DV, Liu A, Buchbinder S, Lama JR, Guanira JV, et al. Emtricitabine-Tenofovir Concentrations and Pre-Exposure Prophylaxis Efficacy in Men Who Have Sex with Men. Sci Transl Med. 2012;4: 151ra125–151ra125.](http://paperpile.com/b/d6r85Y/0Wo9E)

47. [Hall HI, Frazier EL, Rhodes P, Holtgrave DR, Furlow-Parmley C, Tang T, et al. Differences in Human Immunodeficiency Virus Care and Treatment Among Subpopulations in the United States. JAMA Intern Med. 2013;173: 1337–1344.](http://paperpile.com/b/d6r85Y/TYidw)

48. [Rhode Island Integrated Prevention & Care Comprehensive and Statewide Coordinated Statement of Need Plan, CY 2017–2020: State of Rhode Island [Internet]. Executive Office of Health & Human Services, Rhode Island Department of Health; Available:](http://paperpile.com/b/d6r85Y/aRNI2) <http://www.eohhs.ri.gov/Portals/0/Uploads/Documents/HIVAIDS/RI_HIV_IntegratedPlan2017-2021.pdf>

49. [2015 Rhode Island HIV/AIDS Epidemiologic Profile with Surrogate Data [Internet]. Rhode Island Department of Health; 2016 Dec. Available:](http://paperpile.com/b/d6r85Y/dK3uw) <http://health.ri.gov/publications/epidemiologicalprofiles/2015HIVAndSurrogateData.pdf>

50. [Touzard Romo F, Gillani FS, Ackerman P, Rana A, Kojic EM, Beckwith CG. Monitored viral load: a measure of HIV treatment outcomes in an outpatient setting in Rhode Island. R I Med J . 2014;98: 26–30.](http://paperpile.com/b/d6r85Y/gAId7)

51. [2014 Rhode Island HIV/AIDS Epidemiologic Profile with Surrogate Data. Rhode Island Department of Health; 2015 Dec.](http://paperpile.com/b/d6r85Y/SIrhH)

52. [Preexposure Prophylaxis for the Prevention of HIV Infection in the United States - 2014: A Clinical Practice Guideline [Internet]. Centers for Disease Control and Prevention; 2014 p. 499. Available:](http://paperpile.com/b/d6r85Y/maCJh) <https://www.cdc.gov/hiv/pdf/prepguidelines2014.pdf>

53. [Chan PA, Glynn TR, Oldenburg CE, Montgomery MC, Robinette AE, Almonte A, et al. Implementation of Preexposure Prophylaxis for Human Immunodeficiency Virus Prevention Among Men Who Have Sex With Men at a New England Sexually Transmitted Diseases Clinic. Sex Transm Dis. 11/2016;43: 717–723.](http://paperpile.com/b/d6r85Y/QUPTL)

54. [Montgomery MC, Oldenburg CE, Nunn AS, Mena L, Anderson P, Liegler T, et al. Adherence to Pre-Exposure Prophylaxis for HIV Prevention in a Clinical Setting. Datta PK, editor. PLoS One. 2016;11: e0157742.](http://paperpile.com/b/d6r85Y/4swQB)

55. [R Core Team. R: A Language and Environment for Statistical Computing [Internet]. Vienna, Austria: R Foundation for Statistical Computing; 2018. Available:](http://paperpile.com/b/d6r85Y/M1p1K) [https://www.R-project.org/](https://www.r-project.org/)

56. [Wickham H, Francois R, Henry L, Müller K. dplyr: A Grammar of Data Manipulation [Internet]. 2017. Available:](http://paperpile.com/b/d6r85Y/06xuo) [https://CRAN.R-project.org/package=dplyr](https://cran.r-project.org/package=dplyr)

57. [Wickham H, Henry L. tidyr: Easily Tidy Data with “spread()” and “gather()” Functions [Internet]. 2018. Available:](http://paperpile.com/b/d6r85Y/8VhM0) [https://CRAN.R-project.org/package=tidyr](https://cran.r-project.org/package=tidyr)

58. [Wickham H. ggplot2: Elegant Graphics for Data Analysis [Internet]. Springer-Verlag New York; 2009. Available:](http://paperpile.com/b/d6r85Y/HD9DB) <http://ggplot2.org>

59. [Arnold JB. ggthemes: Extra Themes, Scales and Geoms for “ggplot2” [Internet]. 2018. Available:](http://paperpile.com/b/d6r85Y/T5PzD) [https://CRAN.R-project.org/package=ggthemes](https://cran.r-project.org/package=ggthemes)

60. [Wickham H. stringr: Simple, Consistent Wrappers for Common String Operations [Internet]. 2018. Available:](http://paperpile.com/b/d6r85Y/ThsJq) [https://CRAN.R-project.org/package=stringr](https://cran.r-project.org/package=stringr)

61. [Garnier S. viridis: Default Color Maps from “matplotlib” [Internet]. 2018. Available:](http://paperpile.com/b/d6r85Y/qtkMv) [https://CRAN.R-project.org/package=viridis](https://cran.r-project.org/package=viridis)

62. [Winston Chang. extrafont: Tools for using fonts [Internet]. 2014. Available:](http://paperpile.com/b/d6r85Y/mWUDJ) [https://CRAN.R-project.org/package=extrafont](https://cran.r-project.org/package=extrafont)

63. [Auguie B. gridExtra: Miscellaneous Functions for “Grid” Graphics [Internet]. 2017. Available:](http://paperpile.com/b/d6r85Y/Gst32) [https://CRAN.R-project.org/package=gridExtra](https://cran.r-project.org/package=gridExtra)

64. [Gohel D. ReporteRs: Microsoft Word and PowerPoint Documents Generation [Internet]. 2018. Available:](http://paperpile.com/b/d6r85Y/r1ugz) [https://CRAN.R-project.org/package=ReporteRs](https://cran.r-project.org/package=ReporteRs)
