# Supplementary material for: Improving the impact of HIV pre-exposure prophylaxis implementation in small urban centers among men who have sex with men: An agent-based modelling study
Source: PLoS One. 2018 Jul 9;13(7):e0199915. doi: 10.1371/journal.pone.0199915 (PMC6037355; doi:10.1371/journal.pone.0199915)
Supplement: S1 Table — HIV prevalence and incidence at 0% PrEP coverage. (DOCX) [file pone.0199915.s002.docx]

**S1 Table. Age mixing sensitivity analysis. HIV prevalence and incidence at 0% PrEP coverage.**

| **Scenario** | **HIV** | **Cumulative Incidence** | **Incidence Rate** | **Incidence Change**  **(%)** |
| --- | --- | --- | --- | --- |
| Main | 7.4  (6.7, 8.1) | 826  (711, 955) | 3.51  (3.00, 4.08) | - |
| *Age Mixing* |  |  |  |  |
| Mix0.00 | 7.1  (6.3, 7.9) | 777  (659, 902) | 3.30  (2.79, 3.85) | -5.9 |
| Mix0.25 | 7.2  (6.4, 7.9) | 784  (665, 905) | 3.33  (2.81, 3.86) | -5.1 |
| Mix0.50 | 7.2  (6.5, 8.0) | 797  (669, 929) | 3.38  (2.83, 3.96) | -3.5 |
| Mix0.75 | 7.3  (6.6, 8.1) | 815  (701, 928) | 3.47  (2.96, 3.96) | -1.3 |
| **Notes:**  Main, base case from main analysis  SF[*X*], sex frequency scale  PN[X], partner number scale  Mix[*X*], proportion of partner selections that use the age mixing matrix to determine the probability of partnership formation  *HIV*, ending HIV prevalence; *CumInc*, new infections over 10 years; *IR*, incidence rate per 1000 person-years at risk  Incidence change = percent change in 10-year median cumulative incidence relative to Main scenario  *Medians and 95% simulation limits reported* | | | | |
