# Supplementary material for: Improving the impact of HIV pre-exposure prophylaxis implementation in small urban centers among men who have sex with men: An agent-based modelling study
Source: PLoS One. 2018 Jul 9;13(7):e0199915. doi: 10.1371/journal.pone.0199915 (PMC6037355; doi:10.1371/journal.pone.0199915)
Supplement: S2 Table — PrEP impact and efficiency at 15% coverage of HIV-negative MSM. (DOCX) [file pone.0199915.s003.docx]

**S2 Table. Age mixing sensitivity analysis. PrEP impact and efficiency at 15% coverage of HIV-negative MSM.**

| **Scenario** | **NIA** | **PIA** | **PYPAI** |
| --- | --- | --- | --- |
| Main | 218  (121, 307) | 26.2  (14.5, 37.0) | 161  (115, 289) |
| *Age Mixing* |  |  |  |
| Mix0.00 | 202  (106, 294) | 25.9  (13.7, 37.9) | 174  (120, 329) |
| Mix0.25 | 203  (107, 292) | 25.9  (13.6, 37.2) | 174  (121, 327) |
| Mix0.50 | 207  (114, 296) | 26.0  (14.3, 37.2) | 170  (119, 306) |
| Mix0.75 | 211  (115, 305) | 25.9  (14.1, 37.5) | 167  (116, 304) |
| **Notes:**  *Main*, base case from main analysis  *Mix[X]*, proportion of partner selections using the age mixing matrix  *PrEP*, pre-exposure prophylaxis; *MSM*, men who have sex with men; *NIA*, number of infections averted, *PIA*, percentage of infections averted; *PYPAI*, person-years per averted infection  *Medians and 95% simulation limits reported* | | | |
