# Supplementary material for: Improving the impact of HIV pre-exposure prophylaxis implementation in small urban centers among men who have sex with men: An agent-based modelling study
Source: PLoS One. 2018 Jul 9;13(7):e0199915. doi: 10.1371/journal.pone.0199915 (PMC6037355; doi:10.1371/journal.pone.0199915)
Supplement: S3 Table — (DOCX) [file pone.0199915.s004.docx]

**S3 Table. Ten-year summary statistics across PrEP allocation scenarios and coverage levels vs. base case (0% PrEP coverage).**

| **Scenario** | **HIV Prevalence (%)** | **New Infections** | **Incidence Rate*** | **Infections Averted (#)** | **Infections Averted (%)** | **PYPAI** |
| --- | --- | --- | --- | --- | --- | --- |
| *Coverage 0%* | | | | | | |
| No PrEP | 7.4  (6.7, 8.1) | 826  (711, 955) | 3.51  (3.00, 4.08) | - | - | - |
| *Coverage 5%* | | | | | | |
| Current patients | 7.1  (6.4, 7.9) | 754  (639, 874) | 3.20  (2.70, 3.73) | 76  (-44, 191) | 9.1  (-5.3, 23.0) | 154  (61, 11644) |
| Random | 7.1  (6.4, 7.9) | 768  (661, 890) | 3.26  (2.79, 3.80) | 62  (-60, 169) | 7.4  (-7.3, 20.3) | 189  (69, 11653) |
| PN > 5 | 7.0  (6.4, 7.8) | 741  (636, 860) | 3.14  (2.69, 3.66) | 89  (-30, 194) | 10.7  (-3.7, 23.3) | 132  (61, 11634) |
| PN > 10 | 7.0  (6.3, 7.7) | 731  (625, 842) | 3.10  (2.64, 3.58) | 99  (-12, 205) | 11.9  (-1.5, 24.7) | 118  (57, 11616) |
| *Coverage 10%* | | | | | | |
| Current patients | 6.8  (6.1, 7.5) | 681  (572, 786) | 2.89  (2.41, 3.35) | 149  (44, 258) | 17.9  (5.3, 31.1) | 158  (91, 535) |
| Random | 6.9  (6.2, 7.6) | 709  (601, 819) | 3.01  (2.53, 3.49) | 121  (11, 229) | 14.5  (1.3, 27.6) | 194  (103, 2198) |
| PN > 5 | 6.7  (6.1, 7.5) | 664  (571, 778) | 2.82  (2.41, 3.31) | 166  (52, 259) | 20.0  (6.2, 31.2) | 141  (91, 451) |
| PN > 10 | 6.6  (6.0, 7.3) | 635  (545, 739) | 2.69  (2.30, 3.14) | 195  (91, 285) | 23.5  (10.9, 34.3) | 120  (82, 257) |
| *Coverage 15%* | | | | | | |
| Current patients | 6.5  (5.9, 7.2) | 612  (523, 709) | 2.59  (2.21, 3.02) | 218  (121, 307) | 26.2  (14.5, 37.0) | 161  (115, 289) |
| Random | 6.7  (6.0, 7.4) | 654  (546, 756) | 2.77  (2.31, 3.22) | 176  (74, 284) | 21.2  (8.9, 34.2) | 199  (124, 474) |
| PN > 5 | 6.5  (5.8, 7.1) | 595  (499, 691) | 2.52  (2.11, 2.94) | 235  (139, 331) | 28.3  (16.7, 39.9) | 150  (107, 252) |
| PN > 10 | 6.3  (5.7, 6.9) | 555  (478, 639) | 2.35  (2.01, 2.71) | 275  (191, 352) | 33.1  (23.0, 42.4) | 128  (100, 184) |
| *Coverage 20%* | | | | | | |
| Current patients | 6.3  (5.7, 7.0) | 550  (468, 645) | 2.33  (1.98, 2.74) | 280  (185, 362) | 33.7  (22.3, 43.6) | 168  (130, 252) |
| Random | 6.5  (5.8, 7.2) | 602  (507, 699) | 2.55  (2.14, 2.97) | 228  (131, 323) | 27.4  (15.8, 38.9) | 206  (145, 356) |
| PN > 5 | 6.2  (5.6, 6.8) | 530  (450, 612) | 2.24  (1.90, 2.60) | 300  (218, 380) | 36.1  (26.2, 45.8) | 157  (124, 215) |
| PN > 10 | 6.3  (5.7, 6.9) | 540  (462, 626) | 2.28  (1.95, 2.66) | 290  (204, 368) | 34.9  (24.6, 44.3) | 130  (102, 183) |
| *Coverage 25%* | | | | | | |
| Current patients | 6.0  (5.5, 6.6) | 488  (412, 567) | 2.06  (1.73, 2.41) | 341  (263, 418) | 41.1  (31.7, 50.3) | 172  (141, 222) |
| Random | 6.3  (5.7, 7.0) | 553  (470, 652) | 2.34  (1.98, 2.77) | 277  (178, 360) | 33.3  (21.4, 43.4) | 211  (163, 328) |
| PN > 5 | 6.0  (5.4, 6.5) | 470  (398, 548) | 1.98  (1.68, 2.32) | 360  (282, 432) | 43.4  (33.9, 52.0) | 163  (136, 208) |
| PN > 10 | 6.2  (5.7, 6.9) | 537  (457, 633) | 2.27  (1.93, 2.69) | 293  (197, 373) | 35.3  (23.7, 44.9) | 129  (101, 190) |
| *Coverage 30%* | | | | | | |
| Current patients | 5.8  (5.3, 6.4) | 434  (370, 505) | 1.83  (1.56, 2.14) | 395  (325, 460) | 47.6  (39.1, 55.4) | 178  (154, 216) |
| Random | 6.1  (5.5, 6.8) | 503  (427, 589) | 2.13  (1.80, 2.50) | 327  (241, 403) | 39.4  (29.0, 48.5) | 216  (175, 290) |
| PN > 5 | 5.7  (5.2, 6.3) | 408  (345, 472) | 1.72  (1.45, 2.00) | 422  (358, 485) | 50.8  (43.1, 58.4) | 167  (146, 196) |
| PN > 10 | 6.3  (5.6, 6.9) | 540  (461, 631) | 2.29  (1.94, 2.68) | 290  (199, 369) | 34.9  (23.9, 44.4) | 131  (102, 188) |
| **Notes:** *HIV Prevalence*, ending HIV prevalence; *PYPAI*, person-years on PrEP per averted infection; *Current patients*, Current Patient Population scenario; *PN > 5*, expected annual partner number greater than 5; *PN > 10*, expected annual partner number greater than 10  Medians and 95% simulation limits presented. For simulation runs in which a PrEP scenario produced more infections than mean 10-year cumulative HIV incidence at 0% coverage, PYPAI set to the number of person-years on PrEP.  * Incidence rate per 1000 person-years at risk. | | | | | | |
